# Long-Term Effects of Isoflurane Anesthesia and Surgery: Sex-Associated Neurological Dysfunction and Alterations in Circulating Extracellular Vesicles

**DOI:** 10.3390/ijms27219936

**Published:** 2026-11-06

**Authors:** Yun Li, Ruth Park, Balaji Krishnamachary, Hangnoh Lee, Zhuofan Lei, Hui Li, Junfang Wu

**Affiliations:** 1 Department of Anesthesiology and Shock, Trauma and Anesthesiology Research Center (STAR), University of Maryland School of Medicine, Baltimore, MD 21201, USA; 2 Department of Medicine, University of Maryland School of Medicine, Baltimore, MD 21201, USA; 3 University of Maryland- Medicine Institute for Neuroscience Discovery (UM-MIND), University of Maryland School of Medicine, Baltimore, MD 21201, USA

**Keywords:** isoflurane, olfactory deficits, postoperative cognitive dysfunction, long-term neurological outcomes, extracellular vesicles, proteomic

## Abstract

Volatile anesthetics and surgery can trigger neuroinflammation and neurological dysfunction, but their long-term effects remain poorly understood. Young adult male and female C57BL/6 mice underwent 4 h of 2% isoflurane anesthesia combined with laparotomy (Iso/OP) or sham treatment. At 12 weeks, olfactory and cognitive functions were assessed, followed by RNA sequencing of olfactory bulbs and hippocampi and plasma extracellular vesicle (EV) profiling. EV-associated inflammatory bioactivity was evaluated by hippocampal injection into naïve male mice. Iso/OP induced olfactory deficits at 12 weeks, with greater impairment in females. Female mice also exhibited deficits in spatial working memory and associative learning, whereas males showed relatively preserved cognitive function. Transcriptomic analyses revealed sex-dependent alterations in pathways related to synaptic function, neurodevelopment, extracellular matrix remodeling, stress responses, and chemotaxis. Iso/OP reduced circulating EV abundance, particularly among 40–100 nm particles, and altered EV proteomic profiles. Exploratory analyses of pooled hippocampal tissues revealed distinct cytokine and chemokine profiles following injection of plasma EVs from the four donor groups. Collectively, these findings demonstrate sex-dependent neurological and molecular alterations persisting 12 weeks after Iso/OP, accompanied by changes in circulating EVs. The exploratory EV transfer findings warrant further investigation into the potential contribution of circulating EVs to brain inflammatory responses.

## 1. Introduction

Volatile anesthetics remain essential in clinical and experimental anesthesia [1,2], yet growing evidence indicates that anesthesia and surgery can produce persistent neurological effects [3,4,5]. Although sevoflurane has replaced isoflurane (Iso) in many operating rooms, Iso remains widely used in intensive care, resource-limited settings, and preclinical research [6,7,8,9,10]. To maintain consistency with established postoperative neurocognitive dysfunction paradigms, we used an isoflurane plus laparotomy surgery (Iso/OP) model that has been characterized in our previous studies and by others [11,12,13]. Because general anesthesia is almost always accompanied by surgery and systemic inflammation, prolonged Iso exposure combined with laparotomy operation (OP) provides a clinically relevant model to investigate long-term neurological consequences. Although postoperative neurocognitive dysfunction has been studied mainly in older adults [14,15,16,17], prolonged anesthesia is also common in younger patients undergoing major surgery. Clinical perioperative studies report typical major-surgery anesthesia or operating-room durations of approximately 3–4 h, with upper-range values extending beyond 4 h in some surgical cohort [18,19,20]. Thus, the 4 h Iso/OP paradigm models a clinically plausible duration of maintenance anesthesia during prolonged surgery rather than an unusually severe exposure. Using young adult mice further allowed us to examine anesthesia- and surgery-associated neurological changes without the added confounding influence of advanced aging.

Previous studies have shown that Iso/surgery induces acute and subacute cognitive and olfactory dysfunction accompanied by neuroinflammation and synaptic impairment [11,17]. Long-term neurological outcomes are clinically important because anesthesia and surgery may induce persistent changes beyond early postoperative recovery. Although clinical evidence for lasting neurological dysfunction remains mixed [21,22,23], preclinical models allow direct evaluation of long-term behavioral and molecular consequences. The olfactory bulb (OB) and hippocampus (HI), key regions for olfaction, memory, and learning, are particularly vulnerable to perioperative injury [24]. Consistent with our previous transcriptomic studies in aged mice, profiling these regions links persistent behavioral deficits to underlying molecular changes [11,25].

Extracellular vesicles (EVs) have emerged as key mediators of intercellular communication and a potential link between peripheral inflammation and central nervous system (CNS) dysfunction [26]. EVs are membrane-bound nanoparticles that carry proteins, lipids, and nucleic acids reflective of their cells of origin and can cross the blood–brain barrier to deliver bioactive cargo to recipient cells [27,28]. Circulating EVs contribute to neuroinflammatory signaling in neurological disorders [29,30,31], and their abundance and cargo are altered after anesthesia and surgery [25,32,33,34], suggesting a role in postoperative neurological dysfunction. Whether they mediate chronic neuroinflammation after prolonged anesthesia and surgery remains unknown.

Biological sex is a recognized modifier of neurological outcomes after general anesthesia and surgery, but the direction of vulnerability remains inconsistent. Clinical studies have reported higher early postoperative delirium risk in male cardiac surgery and hip fracture patients [35,36], whereas other analyses suggest greater risk in females [37]. Experimental studies show sex-dependent anesthetic effects: female 5xFAD mice developed cognitive deficits after sevoflurane and surgery, whereas neonatal male rats exhibited greater long-term impairment after Iso exposure [38,39]. Sex also affects anesthetic sensitivity during Iso anesthesia in mice [40]. Biological sex may influence circulating EV characteristics, including particle concentration and protein cargo [41]. However, whether prolonged anesthesia and surgery induce sex-dependent alterations in brain transcriptomes and circulating EV-associated inflammatory signaling during the chronic phase remains unclear. This study therefore examined whether sex modifies chronic neurological responses to prolonged Iso/OP. Behavioral outcomes at 12 weeks were compared with age- and sex-matched sham controls, focusing on sex-dependent treatment effects rather than within-animal longitudinal changes.

In the present study, we used a mouse model of prolonged Iso anesthesia combined with laparotomy to examine long-term neurological, brain transcriptomic, and circulating EV changes in young adult male and female mice. We assessed behavioral outcomes and molecular profiles 12 weeks after Iso/OP and explored hippocampal cytokine and chemokine profiles following direct plasma EV injection into naïve male recipients. This approach examined sex-associated chronic responses to Iso/OP and provided an exploratory assessment of hippocampal inflammatory signals following plasma EV transfer.

## 2. Results

### 2.1. Extended Iso/OP Induces Chronic Sex-Dependent Post-Operative Neurobehavioral Deficits

To assess chronic neurological dysfunction, adult male and female C57BL/6 mice underwent abdominal surgery followed by 4 h of Iso anesthesia. A battery of behavioral tests was performed 12 weeks after Iso/OP to evaluate long-term neurobehavioral outcomes. Odor memory was first assessed using the odor recognition test. Mice were initially exposed to a cotton swab soaked in a cinnamon solution, followed by a mildly aversive odor, and they were re-exposed to the same odor at least 1 h later. Mice that successfully remembered the odor displayed reduced investigation during the second trial. Baseline odor memory was comparable between male and female sham mice, with no significant sex differences detected. At 12 weeks after Iso/OP, both male (LS mean difference, −24.47; 95% CI, −44.48 to −4.46; adjusted *p* = 0.0113) and female mice (LS mean difference, −44.72; 95% CI, −65.33 to −24.11; adjusted *p* < 0.0001) exhibited impaired odor memory compared with their respective sham controls; however, no significant sex difference was observed between the Iso/OP groups (LS mean difference, −18.20; 95% CI, −38.21 to 1.81; adjusted *p* = 0.0860; Figure 1A). We next evaluated odor sensitivity using the buried food test; sham male and female mice did not differ significantly (LS mean difference, −27.44; 95% CI, −60.28 to 5.39; adjusted *p* = 0.1296), whereas Iso/OP impaired odor sensitivity in both males (−33.97; 95% CI, −65.85 to −2.09; adjusted *p* = 0.0330) and females (−54.06; 95% CI, −86.89 to −21.22; adjusted *p* = 0.0004), with greater impairment in Iso/OP females than males (−47.53; 95% CI, −79.41 to −15.65; adjusted *p* = 0.0015; Figure 1B). These findings suggest that Iso/OP induced olfactory deficits evident as late as 12 weeks after surgery, with female mice exhibiting greater impairment in odor sensitivity. To assess long-term cognitive function, HI-dependent spatial working memory was evaluated using the Y-maze. Iso/OP/F mice displayed significantly reduced spontaneous alternation compared with Sham/F controls (LS mean difference, 28.32; 95% CI, 3.786 to 52.85; adjusted *p* = 0.0182), whereas no significant impairment was detected in male mice (LS mean difference, 14.80; 95% CI, −9.017 to 38.62; adjusted *p* = 0.3542; Figure 1C,D). Similarly, active avoidance testing, which measures associative learning and memory, revealed a significant reduction in avoidance performance in Iso/OP/F mice but not in Iso/OP/M mice (Figure 1E,F). Collectively, these findings demonstrate that extended Iso/OP induces chronic neurobehavioral deficits in young adult mice. Although both sexes exhibited long-term olfactory impairments, female mice were more susceptible to Iso/OP-induced deficits, displaying greater impairment in odor sensitivity, spatial working memory, and associative learning.

In addition, general locomotor activity and neuromuscular function were assessed (Figure S1). In the open-field test, Sham/F mice traveled a greater total distance than Sham/M mice, indicating a baseline sex difference. However, Iso/OP did not affect any open-field parameters (total distance, maximum speed, mean speed, or immobility time) in either sex. Likewise, rotarod performance and grip strength showed no sex- or Iso/OP-dependent differences. As expected, female mice weighed significantly less than males, but body weight was unaffected by Iso/OP in either sex. Together, these findings indicate that Iso/OP did not alter locomotor activity, neuromuscular function, or body weight under the conditions tested, while expected baseline sex differences were preserved.

### 2.2. Olfactory Bulbs and Hippocampus Undergo Sex-Divergent Transcriptomic Remodeling After Chronic Iso/OP

Because the long-term neurological impairments were primarily localized to the OB and HI, we next examined molecular changes in these regions using bulk RNAseq at 12 weeks after Iso/OP cessation. In the OB, PLS-DA of normalized gene counts showed clear separation among the four groups (Figure 2A). Differential expression analysis (FDR < 0.05) identified only modest transcriptional differences between Iso/OP/F and Iso/OP/M mice, with the most significant DEGs primarily representing sex chromosome-linked genes, including *Kdm5d*, *Ddx3y*, *Uty*, and *Xist* (Figure 2B). Gene Ontology (GO) biological process analysis revealed significant enrichment only among downregulated DEGs (Figure 2C). The top enriched pathways involved small GTPase-mediated signal transduction, synapse organization, and synaptic signaling, with representative genes shown in the accompanying heatmaps and log2 fold-change plots (Figure 2D, E). Reactome analysis further identified cellular stress, the RHO GTPase cycle, and axon guidance as the most significantly regulated pathways (Figure 2F), while WikiPathways highlighted MAPK signaling, mechanisms of pluripotency, and EGFR1 signaling as the principal pathways exhibiting sex-dependent regulation following Iso/OP (Figure 2G).

Because Iso/OP caused olfactory deficits evident 12 weeks after exposure, with greater impairment in female mice, we next examined baseline sex differences and sex-specific transcriptomic remodeling in the OB at this late post-exposure time point. Comparisons were performed between Sham/F and Sham/M, Iso/OP/M and Sham/M, and Iso/OP/F and Sham/F groups. Baseline analysis revealed clear sex-associated differences in OB gene expression, with prominent differential expression of sex chromosome-linked genes, including *Xist*, *Ddx3y*, *Eif2s3x*, *Kdm5d*, and *Uba1y* (Figure S2A). GO analysis identified enrichment of pathways related to responses to external stimuli, vitamin metabolism, and terpenoid metabolism, while heatmap analysis further demonstrated distinct baseline transcriptional profiles between sexes (Figure S2B–E). In female mice, Iso/OP induced relatively few DEGs but prominently altered pathways involved in synaptic vesicle-mediated transport, vesicle organization and fusion, exocytosis, neurotransmitter release, and membrane fusion, together with enrichment of iron transport, estrogen receptor signaling, and purine metabolism (Figure S2F–H). Consistently, genes involved in synaptic vesicle function and neurotransmitter release, including *Snap25*, *Stx1b*, *Atp6v0e2*, *Atp6v1g2*, *Cplx1*, and *Doc2g*, were differentially expressed (Figure S2I,J). In contrast, male mice exhibited a broader transcriptional response to Iso/OP, with a larger number of DEGs and enrichment of pathways associated with peptide and hormone transport, PI3K/PKB signaling, cell migration, protein localization, and chemotaxis (Figure S2K–M). Representative altered genes included *Per2*, *Crhbp*, *Irs2*, *Vgf*, *Slc25a39*, *Dynll1*, and *Camk2n1* (Figure S2N,O). Together, these findings demonstrate baseline sex-specific transcriptional differences in the OB and distinct molecular responses to Iso/OP. Whereas female mice showed selective remodeling of synaptic vesicle and neurotransmitter transport pathways, male mice exhibited broader changes involving transport, PI3K/PKB signaling, and cell migration. These sex-dependent molecular differences may contribute to the greater olfactory impairment observed in female mice.

We next examined HI transcriptomic changes to determine whether the long-term cognitive deficits were accompanied by molecular remodeling in this key learning- and memory-related brain region. PLS-DA revealed clear separation between sham and Iso/OP groups, with distinct clustering of Iso/OP/F and Iso/OP/M samples (Figure 3A). Direct comparison of Iso/OP/F and Iso/OP/M mice identified pronounced sex-dependent differences in hippocampal gene expression, including expected sex chromosome-linked transcripts (e.g., *Xist*, *Ddx3y*, *Kdm5d*, and *Uty*) (Figure 3B). GO analysis showed that genes upregulated in Iso/OP/F versus Iso/OP/M mice were enriched in pathways related to metal ion response, glycerolipid metabolism, oxidative stress, learning, microtubule dynamics, phospholipid catabolism, extracellular matrix organization, and hyaluronan biosynthesis (Figure 3C). Representative genes from these pathways, including *Aqp1*, *Mt2*, *Enpp2*, *Pdgfb*, and *Rgs14*, showed higher expression in the female HI (Figure 3D,E). In contrast, genes downregulated in Iso/OP/F relative to Iso/OP/M mice were enriched in protein folding, nucleocytoplasmic transport, neurogenesis, protein localization, cell projection organization, mRNA metabolism, forebrain development, and endoplasmic reticulum stress pathways (Figure 3F), with representative male-enriched genes including *Hspa1b*, *Hspb1*, *Hspa5*, *Dnajb1*, *Pdia4*, *Xbp1*, *Drd2*, *Sox11*, and *Btg2* (Figure 3G,H). Together, these findings demonstrate that Iso/OP induces hippocampal transcriptomic remodeling evident 12 weeks after exposure, with distinct patterns observed in female and male mice. Female mice exhibited enrichment of upregulated genes associated with oxidative stress, lipid metabolism, extracellular matrix remodeling, and learning-related processes, whereas male mice showed greater enrichment of protein-folding, nucleocytoplasmic transport, neurodevelopmental, and endoplasmic reticulum stress pathways. These molecular differences parallel the sex-specific behavioral impairments observed in spatial working memory and associative learning.

In the HI, comparison of sham female and sham male mice revealed clear baseline sex-dependent transcriptional differences, largely driven by expected sex chromosome-linked transcripts (Figure S3A). GO analysis showed enrichment of pathways related to body fluid regulation, glomerular filtration, astrocyte development, and epithelial/glial cell migration (Figure S3B), while heatmap analysis highlighted distinct baseline expression of genes involved in water transport, extracellular matrix organization, and structural remodeling (Figure S3C). In female mice, Iso/OP induced modest but detectable transcriptomic remodeling relative to sham controls (Figure S3D). Upregulated DEGs were enriched in developmental, gliogenic, dendritic spine, tissue remodeling, and axon guidance pathways (Figure S3E), whereas downregulated DEGs were associated with chemotaxis- and immune-related processes (Figure S3F). Representative genes, including *Fgfr3*, *Shank1*, *Cdk5r2*, *Dlgap4*, *Cst3*, *Plec*, *Shank3*, and *Scg2*, showed altered expression in Iso/OP females (Figure S3G,H). In contrast, Iso/OP produced minimal hippocampal transcriptional changes in male mice, with Top1 as the only significantly upregulated gene (Figure S3I). Accordingly, GO enrichment was limited and driven by a small number of genes, highlighting pathways related to embryonic cleavage, DNA conformation, rRNA transcription, circadian regulation, and responses to cAMP or organophosphorus compounds (Figure S3J). Heatmap analysis confirmed increased Top1 expression in Iso/OP males (Figure S3K). Together, these analyses demonstrate pronounced baseline sex differences in the hippocampal transcriptome and distinct sex-specific responses to Iso/OP. Female mice exhibited broader remodeling involving neurodevelopmental, synaptic, extracellular matrix, and immune pathways, whereas male responses were comparatively limited, supporting the primary findings of sex-dependent long-term hippocampal molecular remodeling after Iso/OP.

To characterize coordinated pathway-level responses to Iso/OP, we performed pre-ranked GSEA using the complete gene lists ranked by the DESeq2 Wald statistics. In the HI, the response to Iso/OP was limited in males, with significant positive enrichment of the unfolded protein response, whereas females exhibited broader pathway-level changes, including positive enrichment of oxidative phosphorylation and negative enrichment of epithelial–mesenchymal transition, the G2/M checkpoint, TNF-α signaling via NF-κB, and the IFN-γ response (Figure S4A; Table S1). Notably, several hippocampal pathways showed nearly opposite enrichment directions between males and females, including the G2/M checkpoint, IFN-γ response, oxidative phosphorylation, TNF-α signaling via NF-κB, UV response, and mTORC1 signaling. The androgen-response signature also showed a clear sex-dependent directional difference. Formal interaction analysis confirmed significant Sex × Iso/OP effects for oxidative phosphorylation (NES = 1.68, FDR q = 0.0008) and the G2/M checkpoint (NES = −1.63, FDR q = 0.0026), with the IFN-γ response approaching the FDR threshold (FDR q = 0.0505). Iso/OP produced more extensive pathway enrichment in the OB in both sexes (Figure S4A; Table S1). In males, prominent positively enriched pathways included oxidative phosphorylation, MYC targets V1, UV response, the unfolded protein response, and PI3K-AKT-mTOR signaling. Females similarly demonstrated strong positive enrichment of oxidative phosphorylation and MYC targets V1, accompanied by negative enrichment of the mitotic spindle and TNF-α signaling via NF-κB. Consistent with these divergent sex-specific responses, 22 Hallmark pathways exhibited significant Sex × Iso/OP interactions in the OB. The strongest interactions included TNF-α signaling via NF-κB, oxidative phosphorylation, androgen response, inflammatory response, and the p53 pathway.

Female-versus-male comparisons further demonstrated that sex-associated pathway enrichment depended strongly on treatment condition (Figure S4B). Under sham conditions, the female HI showed positive enrichment of epithelial–mesenchymal transition and IFN-α and IFN-γ responses, whereas mTORC1 signaling, PI3K-AKT-mTOR signaling, mitotic spindle, and oxidative phosphorylation were enriched in males. Following Iso/OP, females instead showed relative enrichment of myogenesis, epithelial–mesenchymal transition, peroxisomal pathways, xenobiotic metabolism, and oxidative phosphorylation, while males showed relative enrichment of mTORC1 signaling, the G2/M checkpoint, TNF-α signaling via NF-κB, the unfolded protein response, and hypoxia-related genes. A similar treatment-dependent reorganization occurred in the OB: baseline female enrichment of IFN responses and metabolic pathways shifted after Iso/OP toward strong female enrichment of oxidative phosphorylation and lipid metabolism, accompanied by relative male enrichment of TNF-α signaling via NF-κB, inflammatory response, and mitotic spindle pathways. Thus, Iso/OP did not simply amplify baseline sex differences but altered both their magnitude and biological composition across brain regions. Collectively, these findings demonstrate that sex is a major determinant of the brain’s transcriptional response to prolonged Iso/OP and reveal distinct metabolic, inflammatory, cell-cycle, and stress-response programs that may contribute to the sex- and region-dependent neurological outcomes observed in this study.

To assess the relationship between OB and HI remodeling, we compared Hallmark Gene Set Enrichment Analysis (GSEA) patterns across the two regions. Pathway-level responses showed partial concordance between OB and HI, with shared sex-dependent remodeling of oxidative phosphorylation, TNF-α signaling via NF-κB, inflammatory response, IFN-γ response, IL2/STAT5 signaling, androgen response, G2/M checkpoint, mTORC1/PI3K-AKT-mTOR signaling, and hypoxia-related pathways. The concordance was strongest in the Iso/OP female-versus-male comparison, indicating that chronic Iso/OP reorganized sex-associated pathway architecture across both olfactory and hippocampal regions. These findings suggest that the OB and HI responses represent coordinated but regionally specialized components of a broader sex-dependent neurobiological response to chronic Iso/OP.

To further characterize sex-dependent transcriptional responses to ISO/OP, we performed formal sex × treatment interaction analyses in the olfactory bulb and hippocampus (Figure S5). These analyses identified 15 and 21 genes with significant interactions in the olfactory bulb and hippocampus, respectively (FDR < 0.05). In the olfactory bulb, 12 genes exhibited positive interaction effects, indicating greater treatment-associated expression changes in females relative to males, whereas three exhibited negative interaction effects. Similarly, the hippocampus contained 12 genes with positive and nine with negative interaction effects. These findings demonstrate that ISO/OP induces sex-dependent transcriptional responses in both brain regions, with distinct regional patterns of differential treatment responsiveness.

To evaluate the robustness of the transcriptomic group separation identified by PLS-DA, repeated cross-validation and label permutation testing were performed (Figure S6). The hippocampal models achieved mean held-out classification accuracies of 53.1% and 62.5% using two and three latent components, respectively, while the corresponding olfactory bulb models achieved accuracies of 65.0% and 73.1%. All four models substantially exceeded the chance classification level of 25%, with permutation testing confirming significantly greater classification accuracy than randomly shuffled group labels (999 permutations; all *p* = 0.001). These results support reproducible multivariate discrimination among the experimental groups in both brain regions.

### 2.3. Chronic Iso/OP Disrupts Circulating EV Profiles and Protein Cargo

To determine whether Iso/OP exposure and biological sex alter circulating EVs during the chronic post-surgical phase, plasma EVs were prepared 12 weeks after Iso/OP or sham exposure. NanoFCM analysis revealed significantly reduced plasma EV concentrations in both male and female Iso/OP mice compared with their respective sham controls (Figure 4A). Two-way ANOVA further demonstrated that plasma EV concentrations were lower in Sham/F mice than in Sham/M mice and were further reduced in Iso/OP/F mice relative to Iso/OP/M mice (Figure 4B). Size-distribution analysis showed that circulating EVs were predominantly enriched within the small EV range, with peak abundance between 40 and 100 nm (Figure 4C). Across both sexes, Iso/OP reduced particle abundance around this major distribution peak compared with sham controls, with the greatest reduction observed in Iso/OP/M mice. Binned size analysis confirmed that the observed group differences were driven primarily by the 40–100 nm fraction, whereas larger particle populations were less abundant and exhibited greater variability (Figure 4D). NanoFCM analysis confirmed the presence of the EV-associated tetraspanins CD9, CD63, and CD81. The proportions of CD9- and CD81-positive EVs were significantly reduced following Iso/OP/M, whereas CD63-positive EVs were not significantly altered (Figure 4E). Western blot analysis of pooled EV samples showed no detectable calnexin, whereas albumin and ApoB were detected (Figure S7), indicating minimal detectable cellular contamination with residual co-isolation of plasma proteins and lipoproteins in the ultracentrifugation-enriched EV preparations. Collectively, these findings demonstrate that Iso/OP induces chronic, sex-dependent alterations in circulating EV abundance and size distribution, particularly within the small EV-enriched population.

Furthermore, EV-associated proteins were profiled using the Olink Mouse Exploratory panel 12 weeks after exposure. PLS-DA revealed clear separation among the four groups, indicating that both biological sex and Iso/OP exposure contributed to variation in EV protein composition (Figure 5A). Notably, Iso/OP/M and Iso/OP/F samples separated along distinct axes, suggesting divergent EV protein signatures following Iso/OP. Exploratory differential abundance analysis identified multiple EV-associated proteins with nominal *p*-values below 0.05 that differed between Iso/OP/M and Iso/OP/F mice (Figure 5B). Among these, lipoprotein lipase (LPL; FDR-adjusted *p* < 0.05 in both the Iso/OP/Female versus Iso/OP/Male and Iso/OP/Male versus Sham/Male comparisons) was markedly reduced in Iso/OP/F mice relative to Iso/OP/M mice and was the only protein to meet both the statistical-significance and prespecified log2 fold-change≥ 1 criteria. Additional proteins, including CCL2, CCL20, CXCL1, PRDX5, FST (FDR-adjust *p* < 0.05 in Iso/OP/Female vs. Iso/OP/Male), FLI1, WISP1, SNAP29 and Pla2g4a (both met FDR-adjusted *p* < 0.05 in Sham/Female vs. Sham/Male), ADAM23, and PDGFB, also exhibited sex- or condition-dependent differences in EV abundance, with the majority only meeting nominal *p*-value significance unless otherwise noted. Heatmap visualization of group-mean z-scores demonstrated coordinated remodeling of EV protein cargo across sham and Iso/OP groups, with distinct protein clusters enriched in male or female Iso/OP mice (Figure 5C). Representative analyte plots further illustrated sex-dependent abundance patterns for selected EV-associated proteins (Figure 5D). Several inflammatory and signaling molecules, including CCL2, CCL20, CXCL1, and WISP1, displayed differential abundance between male and female Iso/OP mice, whereas LPL, FST, PRDX5, and ADAM23 highlighted broader alterations related to lipid metabolism, oxidative stress, and neurodevelopmental processes. Together, these findings demonstrate that Iso/OP induces sustained remodeling of circulating EV protein cargo, generating distinct sex-dependent molecular signatures that may reflect divergent systemic and neuroimmune responses to anesthetic and surgical stress.

To further investigate whether this sex-dependent remodeling preferentially involved inflammatory signaling, we next focused on a predefined subset of cytokine-, chemokine-, and inflammation-related proteins identified in the EV Olink dataset. PLS-DA based on this subset of inflammatory EV-associated proteins demonstrated clear separation between Iso/OP/M and Iso/OP/F samples, indicating distinct inflammatory EV signatures following Iso/OP (Figure 6A). Heatmap analysis likewise revealed coordinated, sex-dependent differences in inflammatory EV protein cargo across the groups (Figure 6B). Several inflammatory mediators, including CXCL1, CCL2, IL-6, and CCL3, exhibited higher relative abundance in EVs from Iso/OP/M mice, whereas other immune-related proteins showed preferential enrichment in Iso/OP/F mice or sham-derived EVs. These findings suggest that Iso/OP does not globally increase or suppress EV-associated inflammatory proteins but instead selectively reshapes the inflammatory cargo of circulating EVs in a sex-dependent manner. Representative analyte plots further confirmed sex- and condition-dependent differences in selected inflammatory EV-associated proteins (Figure 6C). Collectively, these data demonstrate that Iso/OP produces remodeling of the inflammatory protein landscape carried by circulating EVs that remains evident 12 weeks after exposure. These observations provided the rationale for subsequently testing whether plasma EVs isolated from Iso/OP mice possess functional inflammatory bioactivity capable of modulating inflammatory responses in brain tissue.

### 2.4. Exploratory Profiling of HI Cytokine and Chemokine Responses to Plasma EVs

To explore whether plasma EVs generated after Iso/OP exposure influence hippocampal inflammatory signals, EVs isolated from Sham/M, Sham/F, Iso/OP/M, and Iso/OP/F donor mice were stereotactically injected into the HI of naive adult male C57BL/6 recipients. HI tissues were collected 24 h after injection and pooled within each recipient group for cytokine/chemokine-array analysis. Exploratory analysis showed differences in relative cytokine and chemokine signals across the four donor groups (Figure 7). Nine analytes including PF-4/CXCL4, IGFBP-3, Fractalkine/CX3CL1, IL-12, GM-CSF, MIP-1α/CCL3, LIX/CXCL5, Leptin R, and MCP-5/CCL12 showed higher normalized signals in the Iso/OP/M pool than in the Iso/OP/F pool. Compared with the male sham pool, the Iso/OP/M pool showed higher signals for IL-12, GM-CSF, MIP-1α, and IGFBP-3. Compared with the female sham pool, the Iso/OP/F pool showed lower signals for IGFBP-3, Fractalkine, and MCP-5. These descriptive patterns suggest that hippocampal responses to plasma EVs may vary with donor sex and Iso/OP exposure. Because each condition was represented by one pooled tissue sample, these findings are exploratory and were evaluated without inferential statistical comparisons. Together, these descriptive findings suggest that plasma EVs from donors differing in sex and Iso/OP exposure may elicit distinct acute hippocampal cytokine and chemokine responses. Confirmation of these patterns requires analysis of independent biological samples.

## 3. Discussion

The present study demonstrates that Iso/OP exposure is associated with long-term neurological and systemic alterations evident beyond the perioperative period. At 12 weeks after exposure, Iso/OP mice exhibited olfactory deficits, with greater impairment in females and additional female-associated deficits in odor sensitivity, spatial working memory, and associative learning. These behavioral findings were accompanied by hippocampal and olfactory bulb transcriptomic remodeling, as well as reduced circulating plasma EV abundance and sex- and condition-associated differences in EV protein and inflammatory cargo. Exploratory profiling of pooled recipient tissues showed different HI cytokine and chemokine signals following direct plasma EV injection. Together, these findings identify Iso/OP as a sustained systemic stressor associated with sex-dependent behavioral, molecular, and circulating EV inflammatory changes. The experimental timeline and the relationship between the main donor cohort and the independent EV-transfer recipient cohort are illustrated in Figure 8.

Postoperative neurological dysfunction has primarily been examined during acute or subacute recovery, with many preclinical studies assessing outcomes from hours to several weeks after anesthesia or anesthesia/surgery [42,43,44,45]. Whether prolonged volatile anesthetic exposure combined with surgery produces behavioral abnormalities lasting for months in young adult wild-type mice has remained less clear. This distinction is important because early behavioral changes may reflect transient anesthetic effects, perioperative inflammation, pain, or stress, whereas deficits persisting to 12 weeks suggest more durable neurobiological remodeling. Although the present paradigm was not designed to model intensive care unit sedation directly, prolonged inhaled Iso has been used in critical care sedation through anesthetic-conserving delivery systems [7,46]. Thus, prolonged Iso exposure combined with laparotomy provided a clinically relevant framework to assess neurological outcomes evident 12 weeks after extended anesthesia and surgical stress and to compare these outcomes between male and female mice.

Prior studies have shown that volatile anesthetics and surgery can impair cognition, but they vary widely in animal age, sex, exposure duration, follow-up interval, and behavioral endpoints. Many have focused on short-term outcomes, aged animals, male-only cohorts, or disease-prone models. Iso exposure has been reported to induce hippocampal injury, neuroinflammation, and cognitive impairment in adult mice [42], while exposure concentration and duration can influence later cognitive performance [43,44]. Other studies indicate that age strongly modifies postoperative cognitive outcomes, with aged mice showing greater vulnerability after Iso exposure or surgery than adult mice [47]. In contrast, the role of biological sex has remained less well-defined. The present study addresses this gap by demonstrating that extended Iso/OP induces long-term neurological and behavioral deficits in young adult mice in a sex-dependent manner.

The olfactory findings build on evidence linking postoperative olfactory dysfunction with cognitive impairment. Elderly patients undergoing abdominal surgery with general anesthesia showed olfactory impairment and cognitive decline during early postoperative recovery, with olfactory impairment correlating with memory deficits [48]. Similarly, anesthesia/surgery induced both olfactory and cognitive deficits in mice, which were alleviated by odor enrichment [49]. Sex differences in olfactory function are also well documented, with females generally exhibiting greater odor sensitivity and discrimination than males, although these effects vary by species and experimental conditions [50,51,52,53]. Extending these observations, our findings show that both sexes developed long-lasting olfactory impairment after Iso/OP, while females also developed broader deficits in odor sensitivity, spatial working memory, and associative learning. These results suggest that biological sex influences the chronic neurological consequences of perioperative stress.

The olfactory deficits were accompanied by transcriptomic remodeling in the OB evident 12 weeks after exposure, suggesting that late behavioral impairment is associated with molecular changes in olfactory sensory circuit organization and signaling. Several altered pathways have direct relevance to odor processing. MAPK/ERK signaling contributes to olfactory sensory neuron survival and odor-guided behavior, and ERK5 deletion reduces odor-detection sensitivity and short-term odor memory [54,55]. Although RNAseq was performed in OB tissue rather than olfactory epithelium, Iso/OP-associated changes in MAPK-related pathways may reflect altered glomerular afferent signaling, local OB circuit adaptation, or downstream odor-processing mechanisms. Rho-related signaling, axon guidance, cell projection organization, and synaptic vesicle pathways may also be relevant because olfactory sensory axons converge onto discrete glomerular targets and granule-cell-mediated inhibition of mitral cells is critical for accurate odor discrimination [56,57,58]. Together, these findings suggest that Iso/OP-induced olfactory dysfunction, particularly the greater impairment observed in females, may involve disruption of OB pathways governing synaptic communication, glomerular organization, and activity-dependent sensory processing.

The HI transcriptomic findings provide molecular context for the sex-dependent cognitive deficits observed after chronic Iso/OP. Hippocampal function depends on coordinated regulation of synaptic plasticity, extracellular matrix (ECM) remodeling, metabolism, and neuroimmune signaling, and hippocampal plasticity is sexually dimorphic [59]. ECM remodeling regulates dendritic spine stability and memory-related circuit plasticity [60], while inflammatory chemokines can directly impair hippocampal function. For example, MIP-1α/CCL3 suppresses hippocampal synaptic transmission and LTP, leading to deficits in spatial and long-term memory [61]. Thus, the broader transcriptomic response in Iso/OP females likely reflects coordinated disruption of synaptic, ECM, metabolic, and immune pathways that may contribute to greater cognitive vulnerability.

GSEA revealed coordinated transcriptional responses that differed by sex and brain region, extending the DEG-based findings. The nearly opposite enrichment patterns in male and female HI across metabolic, inflammatory, cell-cycle, and stress-response pathways suggest sex-specific adaptations to Iso/OP, while the broader OB response indicates effects beyond hippocampal pathways. Enrichment of TNF-α/NF-κB and interferon-related gene sets is consistent with prior evidence of perioperative neuroinflammation [47,62,63]. However, GSEA does not establish pathway activation, cellular origin, or causality, and these pathways warrant further mechanistic investigation. Partial concordance between OB and HI GSEA profiles suggests a coordinated, sex-dependent brain response to chronic Iso/OP, linking olfactory and cognitive dysfunction through shared metabolic, inflammatory, and stress-related pathways. The incomplete overlap also indicates region-specific responses, likely reflecting differences in local circuitry, cell composition, and sex-dependent biology. This pattern may contribute to the broader behavioral vulnerability observed in females across olfactory and hippocampal-dependent tasks.

Iso/OP also produced chronic remodeling of circulating plasma EVs, extending the findings from brain-region-specific molecular changes to systemic extracellular signaling. Because EVs were collected 12 weeks after Iso/OP, these changes are unlikely to represent only acute perioperative debris or transient postoperative inflammation. Prior studies show that anesthetics and surgery can alter EV composition and function [64], surgical trauma can modify circulating EV cargo in mice [65], and altered circulating EV cargo has been associated clinically with cognitive decline after major surgery [66]. Our prior work also supports the concept that CNS insults can generate circulating EVs with neuroinflammatory potential [30,67,68], and that Iso/OP induces age-dependent systemic vulnerability with EV protein cargo enriched for inflammatory and growth factor-related signatures in older mice [25]. Consistent with this framework, the present study shows that Iso/OP reduces plasma EV concentration, alters the small EV-enriched size range, and reshapes EV-associated protein and inflammatory cargo in a sex-dependent manner during the chronic phase. Baseline EV abundance was also higher in Sham males than Sham females, consistent with recent human plasma studies showing sex-associated differences in EV particle concentration and proteomic profiles [41]. The mechanisms underlying reduced circulating EV levels remain unclear and may involve altered EV biogenesis, tissue uptake, or systemic clearance.

EV characterization and cargo profiling alone cannot establish whether persistent circulating alterations have functional consequences within the brain. The hippocampal injection experiment explored cytokine and chemokine profiles following administration of plasma EVs collected 12 weeks after Iso/OP. Exploratory profiling identified differences in hippocampal cytokine and chemokine signals across the pooled recipient groups. These observations can be considered alongside previous evidence that anesthesia/surgery-derived EVs induced delirium-like behavioral deficits and neuronal pathology in aged mice [69]. In our previous SCI study, post-ultracentrifugation plasma supernatants did not produce the inflammatory responses observed with SCI plasma EVs in glial cultures [67]. These findings support the rationale for the transfer experiment, although contributions from co-isolated components in the current Iso/OP samples cannot be excluded. Previous studies also indicate that biological sex can influence EV abundance and molecular cargo and that aging may reshape brain EV secretion in a sex-dependent manner [70,71]. In the present study, the descriptive differences in PF-4/CXCL4 and IGFBP-3 signals may implicate platelet/vascular-associated and growth factor-related signaling, whereas Fractalkine/CX3CL1, IL-12, GM-CSF, MIP-1α/CCL3, LIX/CXCL5, Leptin R, and MCP-5/CCL12 are associated with neuroimmune communication, myeloid activation, chemotaxis, and metabolic-inflammatory pathways. These functional associations provide context for the observed signals but do not establish activation of these processes. Because HI tissues were pooled within groups, the cytokine-array analysis could not assess variability among individual recipients. The heatmap provides an exploratory profile of the measured pools, and independent biological samples are needed to establish the reproducibility of these patterns. The EV-transfer experiment examined HI cytokine profiles following direct administration of plasma EVs. This approach bypasses transport from the circulation and therefore does not establish physiological entry of circulating EVs into the brain. It also does not determine whether EVs are required for the chronic behavioral changes or whether the observed cytokine profiles contribute to those changes. The transfer findings are therefore interpreted as exploratory observations that motivate further investigation of the relationship between circulating EVs and brain inflammatory responses. Moreover, because all recipient mice were naïve males, the observed hippocampal cytokine responses are most likely attributable to donor sex-dependent differences in EV bioactivity rather than recipient sex. These findings are therefore limited to male recipients and do not establish whether similar responses occur in females.

Plasma EVs are heterogeneous, originating from platelets, blood and vascular cells, and potentially the CNS under inflammatory conditions [72,73]. Our previous studies demonstrated associations between circulating EV profiles and CNS molecular and inflammatory responses following injury or anesthesia and surgery [25,30,74]. However, the cellular origins of EVs and the surface versus intravesicular localization of detected proteins were not determined, limiting interpretation of their potential biological functions [75,76]. Additionally, electron microscopy was not performed, precluding ultrastructural assessment of EV morphology and heterogeneity [77]. Future studies incorporating ultrastructural imaging, cell-of-origin tracing, and protein localization analyses are warranted. Given the detectable albumin and ApoB, the current proteomic findings should be interpreted as reflecting EV-enriched plasma preparations rather than exclusively EV-derived proteins.

The mechanisms underlying the sex-dependent phenotypes remain unresolved and may involve sex chromosomes, developmental and epigenetic factors, and gonadal hormones [78,79]. Thus, the transcriptomic findings should be viewed as pathway-level signatures associated with behavioral phenotypes rather than evidence of individual mechanistic drivers. Although selected DEGs were not independently validated, the behavioral, transcriptomic, and EV proteomic findings provide complementary evidence of long-term changes evident 12 weeks after Iso/OP, while the EV-transfer findings remain exploratory.

A limitation of this study is the use of young adult mice, which may limit generalizability to older populations relevant to postoperative neurocognitive disorders. Aging involves sex-dependent changes in endocrine function, neuroimmune responses, and EV biology [70,71,80,81], and our previous Iso/OP studies demonstrated age-dependent differences in neurological outcomes, transcriptomic remodeling, and circulating EV profiles [11,25]. Age-by-sex interactions warrant a dedicated, adequately powered study across adult, middle-aged, and aged cohorts. A further limitation is the lack of direct monitoring of arterial blood gases, SpO_2_, end-tidal CO_2_, and blood pressure during the 4 h Iso/OP procedure, as well as the absence of an Iso-only control group in the primary sex-difference cohort. Consequently, the individual contributions of isoflurane anesthesia and surgical stress cannot be fully distinguished, and transient physiological disturbances cannot be excluded despite supplemental oxygen, temperature maintenance, and anesthetic depth monitoring. Although prior studies support adequate oxygenation during oxygen-supported Iso anesthesia in mice [82,83,84,85], future studies incorporating Iso-only and surgery-only controls with continuous physiological monitoring are needed to define their respective contributions. Finally, targeted orthogonal follow-up of selected differentially expressed genes was not performed. Although the RNAseq analyses were designed primarily to identify transcriptome-wide, sex-dependent pathway alterations, targeted molecular analyses could provide additional insight into specific genes contributing to these pathway-level changes.

## 4. Materials and Methods

### 4.1. Animals and Isoflurane/Surgery (Iso/OP) Model

All experimental procedures were approved by the Institutional Animal Care and Use Committees of the University of Maryland School of Medicine (IACUC# AUP-00000092). Adult (10–12-week-old) male (M) and female (F) C57BL/6 mice were obtained from The Jackson Laboratory (Bar Harbor, ME, USA). All mice were housed in the same room of the animal care facility at the University of Maryland School of Medicine under a 12 h light/dark cycle, with ad libitum access to food and water. General anesthesia was induced and maintained with 2% Iso in 100% oxygen for 4 h as previously described [11,25,86]. Anesthesia depth was monitored through respiratory parameters (rate and depth) and reflex responses (palpebral and pedal). Core body temperature was maintained using a heated pad throughout the procedure. Following induction, mice were transferred to a surgical station with a nose cone for continuous Iso maintenance at 2% concentration. A midline laparotomy was performed by making a 1.5 cm incision from the xiphoid process to 0.5 cm above the pubic symphysis, sequentially penetrating the skin, abdominal musculature, and peritoneum. Following surgery, the incision site was treated with 0.25% bupivacaine in sterile saline, then closed in layers using 5-0 monofilament nylon sutures. The surgical procedure lasted approximately 10 to 15 min, after which mice were returned to the anesthesia chamber to complete the 4 h Iso exposure. During recovery, animals were placed on a temperature-regulated pad for 30–60 min to stabilize core temperature. Postoperative monitoring included intensive observation for 4 h post-Iso/OP and daily assessments thereafter to check on general well-being, incision site recovery and hydration. Control animals remained in their home cages and were exposed to room air for 4 h, to better model clinically relevant baseline conditions. Sample sizes were guided by a priori power calculations from our previous animal behavioral studies and experience with comparable experimental paradigms. Before surgery, animals were screened for general health, age- and sex-appropriate body weight, and overt injuries. Only healthy animals meeting these criteria were included, and no animals were excluded during the study. Treatment allocation was randomized at the cage level, with cages assigned to Sham or Iso/OP groups within each sex. All mice within a cage received the same treatment, and animals from different treatment groups were housed separately.

### 4.2. Neurological Function Assessment

Following Iso/OP, a battery of neurological behavioral tests was conducted at the endpoint time of 12 weeks to assess long-term outcomes after exposure. The researcher conducting the behavioral experiments was blinded to group assignments until all behavior experiments were completed. The study included four groups: Sham/Male (*n* = 12), Sham/Female (*n* = 9), Iso/OP/Male (*n* = 10), and Iso/OP/Female (*n* = 12).

**Odor memory (OM) test**: To assess olfactory learning and memory, mice were individually housed overnight and tested using a previously established method [87,88]. Briefly, a clean, dry, cotton-tipped wooden applicator (6 inches long) was inserted through a hole in the cage lid, allowing the mouse to familiarize itself with the applicator for 30 min. A fresh applicator was used for each subject. Cinnamon powder (McCormick & Company, Hunt Valley, MD, USA; 100 ng/ml) was dissolved in water and stored in tightly sealed vials when not in use. During the first trial (T1), the mouse was exposed to the novel odor stimulus. The cotton tip of the applicator was dipped into the odor solution for 2 s before being inserted through the cage lid to a depth of approximately 2.5 cm. The total time the mouse spent sniffing the tip during the 5 min trial (only when oriented toward the tip with its nose within 2 cm) was recorded. At the end of the trial, the applicator was removed. After a 60 min rest period, the second trial (T2) was conducted under the same conditions. The relative sniffing time ratio between trials, calculated as [T2/T1] ×100%, was used to assess memory retention. A reduced sniffing time during T2 indicated recognition and memory of the familiar odor.

**Buried food (BF) test**: To assess olfactory function, the BF test was performed to evaluate the ability of mice to detect and locate a familiar food item hidden beneath the bedding. This test measures the latency of an animal to find a buried, visually inaccessible food pellet using olfactory cues [88]. Briefly, mice were individually housed with ad libitum access to water and food-deprived for 24 h before testing to increase motivation for food seeking. The night before testing, a mini cookie was placed in each home cage for odor familiarization, and consumption was confirmed the following morning to verify palatability. On the test day, each mouse was placed in a clean test cage (46 cm length × 23.5 cm width × 20 cm height) containing 3 cm of fresh bedding and allowed to acclimate for 10 min before returning to its home cage. A uniformly sized mini cookie (~1 g) was then buried 2–3 cm beneath the bedding in a randomly selected corner of the test cage. After the bedding surface was smoothed, the mouse was reintroduced into the test cage, and the latency to locate and begin consuming the buried cookie was recorded. The test was terminated if the mouse failed to locate the cookie within 15 min.

**Open Field:** The open-field test was used to evaluate spontaneous locomotor activity and anxiety-like behavior under dim-light conditions [11,89]. At the beginning of the test, each mouse was placed individually in a corner of a 40 × 40 cm open-field arena, facing the wall, and allowed to explore freely for 5 min. Using the ANY-maze tracking system, the arena was divided into 10 × 10 cm grid squares. The peripheral 10 cm wide area was designated as the outer zone, whereas the central 20 × 20 cm area was defined as the inner zone. Total distance travelled, average and maximum speed, and time spent in the inner and outer zones were recorded and analyzed using ANY-maze software version 7.6 (Stoelting Co., Wood Dale, IL, USA).

**Rotarod Test:** Motor coordination and locomotor function were assessed using an accelerating Rotarod apparatus (Harvard Apparatus, Holliston, MA, USA), as described previously [90,91]. Each mouse was placed on the stationary rod before the start of the trial. The Rotarod accelerated from 4 to 40 rpm over 90 s, with a maximum trial duration of 300 s. The latency to fall from the rotating rod was recorded for each trial. Each mouse completed five trials, and the mean latency to fall was calculated and used for between-group comparisons.

**Grip Strength:** Grip strength was assessed to evaluate neuromuscular function, particularly forelimb muscle strength, using a digital grip strength meter (Ugo Basile, Gemonio, Italy) according to our established protocols [90,91]. Briefly, the forelimb GS of both paws was assessed by placing the mouse on a mesh wire grid connected to the device’s force transducer. Once the mouse secured a firm grip, it was gently held by the tail and slowly pulled away from the grid. The maximum force exerted on the mesh wire grid was recorded, with each mouse undergoing an average of 10 daily trials. Final GS values were normalized to body weight for comparison between groups.

**Y maze (YM) test**: The Y-maze test was performed to assess HI-dependent spatial working memory in mice, as described previously [74,91,92]. The Y-maze apparatus (Stoelting Co.) consisted of three arms of identical length and width, designated A, B, and C. During the test, one arm was randomly selected as the starting position, and each mouse was placed in the maze and allowed to explore freely for 5 min. Arm entries were recorded, and a spontaneous alternation was defined as consecutive entries into three different arms. The percentage of spontaneous alternation was calculated using the following equation: total alternations × 100/(total arm entries − 2). Mice with alternation percentages significantly above 50%, the chance level for entering a novel arm, were considered to have intact spatial working memory.

**Active Avoidance test**: Associative learning was evaluated using a revised version of the two-way active avoidance task in a Gemini shuttle box apparatus (San Diego Instruments, San Diego, CA, USA), as described by Macheda et al. [93]. Mice were moved to the testing room and allowed to acclimate for at least 30 min before testing. The shuttle box consisted of two compartments separated by an open guillotine door. At the beginning of each session, each mouse was placed into one compartment and allowed to habituate freely for 5 min. Each daily session consisted of 30 trials. During each trial, a conditioned stimulus (house light, 10 s) was presented, followed by an unconditioned stimulus (0.2 mA foot shock, 2 s) unless the mouse crossed to the opposite compartment during the CS period. The intertrial interval was randomly varied between 25 and 35 s, with an average interval of 30 s. Crossings during the CS period were scored as avoidance responses, whereas crossings after shock onset were scored as escape responses. Trials in which mice failed to cross during the shock period were scored as escape failures. Avoidance responses, escape responses, and escape failures were quantified to assess associative learning performance. The chamber floor and walls were cleaned between mice to maintain consistent testing conditions.

### 4.3. RNA Extraction and Bulk RNA Sequencing (RNAseq)

Following neurological functional assessment, mice were euthanized, blood samples were collected, and the mice were perfused with 50 mL of ice-cold saline. Total RNA was isolated from dissected OB and HI tissues of mice using the RNAeasy mini kit (Cat# 74106, Qiagen, Hilden, Germany). The RNA samples were sent to Psomagen (Rockville, MD, USA) for mRNA library preparation with poly(A) enrichment. Libraries were prepared for bulk mRNA sequencing and sequenced on an Illumina NovaSeq X Plus platform using paired-end 150 bp reads. Base calls were generated with CASAVA v1.8.2 (Illumina, San Diego, CA, USA). We aligned RNAseq reads to the mouse reference genome (GRCm39) using STAR (version 2.7.5) [94] with GENCODE gene annotation (version M33). Gene and isoform expression levels were quantified in transcripts per million (TPM) using RSEM (version 1.3.3) [95]. Differential expression gene (DEG) analysis was performed with DESeq2 (version 1.42.1) [96] and false discovery rates (FDR) were calculated using the Benjamini–Hochberg method. Genes with Benjamini–Hochberg FDR <0.05 were considered differentially expressed. No minimum absolute log2FC threshold was imposed; log2FC values were retained and reported as continuous measures of effect magnitude. For partial least squares discriminant analysis (PLS-DA), the package Mixomics was used [97]. Genes with an FDR of less than 0.05 were differentially expressed and used for downstream pathway enrichment analysis. Gene ontology (GO) analysis was performed with clusterProfiler 4.10.1 [98]. Gene set enrichment analysis (GSEA) was also performed using clusterProfiler with genes ranked by DESeq2-derived test statistics to identify coordinated pathway-level changes across the full expression dataset. Full GSEA results are provided in Supplementary Table S1.

To identify genes exhibiting sex-dependent transcriptional responses to ISO/OP, differential expression analyses were performed separately for the olfactory bulb and hippocampus using a factorial model incorporating sex, treatment, and their interaction. The interaction effect was defined as the difference between the female treatment response (female ISO/OP versus female sham) and the male treatment response (male ISO/OP versus male sham). Positive interaction coefficients indicated greater treatment-associated log2 fold changes in females, whereas negative coefficients indicated greater changes in males. Statistical significance was determined using the Benjamini–Hochberg false discovery rate (FDR) correction, with FDR < 0.05 considered significant. Interaction coefficients and adjusted *p* values were visualized using volcano plots.

The robustness of the partial least squares discriminant analysis (PLS-DA) models was evaluated separately for the hippocampal and olfactory bulb RNAseq datasets using repeated stratified four-fold cross-validation (five repeats). Models containing two or three latent components were assessed to evaluate the classification performance of the displayed two-component score plots and the originally fitted three-component models, respectively. Classification accuracy was calculated using held-out samples and averaged across cross-validation folds and repeats. To determine whether the observed classification performance exceeded that expected from random group assignments, 999 label permutations were performed for each model, with cross-validation repeated after each permutation. Empirical permutation *p* values were calculated by comparing the observed mean cross-validated accuracy with the corresponding distribution of accuracies obtained from permuted labels. Multiple-testing correction was performed using the Holm method.

### 4.4. Plasma EV Isolation

Blood was collected into precoated EDTA tubes (Cat #365974, BD Biosciences, San Jose, CA, USA) through terminal cardiac puncture under anesthesia. EVs were isolated using differential ultracentrifugation, with minor modifications as described in our previous publications [25,30,67,74]. Briefly, blood samples were immediately centrifuged at 800× *g* for 10 min, followed by 2500× *g* for 10 min, to generate platelet free plasma. To separate larger EVs, equal volumes of plasma from each animal were centrifuged at 12,000× *g* for 30 min at 4 °C. The supernatant was then subjected to ultracentrifugation at 110,000× *g* for 2 h at 4 °C to isolate small EVs. The pellet was washed with phosphate-buffered saline (PBS) and re-centrifuged under the same conditions. The final EV pellet was resuspended in an equal volume of PBS.

### 4.5. Nano Flow Cytometry

Particle concentration and size of EVs were measured using a Flow NanoAnalyzer (NanoFCM Inc., Xiamen, China) [25]. The instrument was calibrated using QC and size standard beads. EV samples were diluted in sterile, molecular-grade water, and the events were recorded for 1 min. Particle concentration and size were calculated using the calibration curves [99]. To detect canonical tetraspanin markers on EVs, 5 μL of EV sample was incubated with APC-conjugated antibodies against CD63 (1:10, Cat.143905, BioLegend, San Diego, CA, USA), CD9 (1:10, Cat.124812, BioLegend), or CD81 (1:10, Cat.104909, BioLegend). For CD9 and CD63, 3 μL of diluted antibody was used, whereas 1 μL was used for CD81. Samples were incubated for 30 min at 37 °C at 60 RPM, followed by dilution in molecular-grade water and acquisition on a NanoFCM instrument. Molecular-grade water alone and antibody-only preparations were used as blank and staining controls, respectively. A total of 2000–12,000 events were recorded per sample. Particle concentration was calculated from flow rate and side-scatter intensity using NanoFCM Profession software (v3.0).

### 4.6. Western Blot Analysis

For Western blot analysis of EV preparation purity, pooled plasma EV samples were lysed and 5 µg of EV protein was loaded per lane. Membranes were probed with primary antibodies against calnexin (mouse monoclonal, Cat. 66903-1-Ig, Proteintech, Rosemont, IL, USA), ApoB (rabbit monoclonal, Proteintech, Cat. 84047-1-RR), and albumin (rabbit antibody, Cat. 4929S, Cell Signaling Technology, Danvers, MA, USA).

### 4.7. Plasma EVs Proteomics and Data Analysis

Plasma EVs were lysed in radioimmunoprecipitation assay buffer for protein quantification. Equal amounts of EV protein (0.5 μg/μL) were loaded in randomized order into a 96-well plate and submitted to Psomagen (Rockville, MD, USA) for proteomic analysis as described in our previous publication [67]. EV protein cargo was profiled using the Olink Mouse Exploratory Panel. Protein detection was performed using Olink’s proximity extension assay, a multiplex immunoassay in which pairs of oligonucleotide-labeled antibodies bind to their target proteins. Upon dual binding, the oligonucleotides hybridize and form a DNA template that is subsequently quantified by real-time PCR. Samples were randomly plated, and assay performance was monitored using internal quality controls. Protein abundance was reported as normalized protein expression (NPX) values on a log2 scale, with higher NPX values indicating higher relative protein levels. Following the manufacturer’s guidelines, samples that did not meet the technical specifications of the assay were excluded from further analysis. For analysis focused on inflammatory EV cargo, we used a predefined subset of proteins measured by the Olink Mouse Exploratory Panel. This subset included cytokines, chemokines, and other inflammation-related signaling proteins selected according to established functional annotations in the Kyoto Encyclopedia of Genes and Genomes (KEGG) and prior publications [67,100,101]. Selection of this subset was determined independently of the observed protein abundance, differential-expression statistics, nominal *p*-values, and FDR-adjusted *p*-values. The subset was used for the inflammatory cargo analyses shown in Figure 6, including PLS-DA and heatmap visualization. Differential protein abundance was analyzed using the limma framework in R, fitting linear models to log2-transformed NPX values followed by empirical Bayes moderation to obtain moderated statistics [102,103]. *p*-values were adjusted for multiple comparisons using the Benjamini–Hochberg false discovery rate (FDR) method. Because relatively few proteins met the FDR-adjusted significance threshold, exploratory visualization of differential protein abundance was performed using nominal *p*-values (*p* < 0.05), with fold-change thresholds applied where indicated. Volcano plots display nominal *p*-values, whereas heatmaps illustrate relative protein abundance using standardized z-scores. Findings based on nominal *p*-values were considered exploratory rather than statistically significant after multiple-testing correction. Complete limma results, including log2 fold changes, nominal *p*-values, and FDR-adjusted *p*-values, are provided in Supplementary Table S2.

### 4.8. Processing of Donor Plasma EV, Intra-Hippocampal Injection, and Cytokine Array

The donor plasma EV samples were pooled within each group and stored at −80 °C until required for injections. For the intra-hippocampal EV injection experiments, adult C57BL6 male mice (24–26 weeks old), age-matched to the donor mice at the experimental endpoint, were anesthetized by Iso and placed in a stereotaxic setup. Artificial eye ointment was applied to protect the eye, and Bupivacaine was injected subcutaneously as local analgesia. Following a midline scalp incision, two small holes were drilled on the skull above the left HI for each mouse (dorsal HI: AP −2.0 mm, ML −1.5 mm, DV 2.0~1.8 mm; Ventral HI: AP −3.0 mm, ML −2.8 mm, DV 3.0~2.8 mm). Plasma EVs were adjusted to approximately 9 × 10^7^ measured particles/µL. Each recipient received 0.5 µL at each of two HI sites through a 5 µL syringe (Hamilton Neuros 700 Series; Hamilton Company, Reno, NV, USA), corresponding to approximately 9 × 10^7^ particles per mouse. The injection volume and flow rate were controlled as 0.5 μL at 0.1 μL/min. The needle was moved up 0.1 mm per 0.25 μL during the injection and was kept in place for 3 additional min and then slowly withdrawn. The mice were sutured with Bupivacaine, reapplied and then returned to their home cages for recovery. All animals were sacrificed for HI tissue collection 24 h later. For cytokine profiling, hippocampal tissues from each group were lysed using the lysis buffer provided in the Mouse Cytokine Array C3-(AAM-CYT-3-8, Ray Biotech). HI tissue lysates were pooled within each recipient group, yielding one tissue pool per condition. Aliquots of the same pool, containing 500 µg of protein per membrane, were analyzed on two separate membranes using the Mouse Cytokine Array C3 according to the manufacturer’s protocol, including overnight sample incubation at 4 °C. Each analyte was spotted in duplicate on each membrane, yielding four technical measurements per analyte per condition. Membrane images were analyzed using Bio-Rad Image Lab software Version 6.1.0 build 7 (Bio-Rad Laboratories, Hercules, CA, USA). Spot intensities were first normalized to the positive control spots on each membrane and then to the male sham EV-injected group. The cytokine-array results were summarized descriptively. For visualization, normalized values were converted to row-scale z-scores for each analyte.

### 4.9. Statistical Analysis

All quantitative data are displayed as individual data points in column graphs, presented as mean ± SEM. Statistical analyses were conducted using GraphPad Prism (version 9.5.0 for Windows, GraphPad Software; RRID: SCR_002798) for all tests. The Shapiro–Wilk test was used to assess data distribution normality. For multiple group comparisons, one-way ANOVA or repeated-measures two-way ANOVA were performed, followed by Tukey’s or Newman–Keuls post hoc tests for parametric data (when normality and equal variance assumptions were met). Detailed statistical analyses for each assay are provided in the figure legends, with a significance threshold set at *p* ≤ 0.05.

## 5. Conclusions

This study identifies extended Iso anesthesia combined with laparotomy as a durable systemic and neurobiological stressor that induces chronic, sex-associated neurological alterations in young adult mice. Twelve weeks after exposure, Iso/OP resulted in sustained olfactory dysfunction, female-biased impairment in hippocampal-dependent behaviors, sex-specific transcriptomic remodeling in the OB and HI, and long-lasting changes in circulating plasma EV abundance and inflammatory cargo. Exploratory analysis of pooled recipient hippocampal tissues showed different cytokine and chemokine profiles following direct administration of plasma EV preparations from the four donor groups. Collectively, these findings suggest that biological sex may influence neuroimmune responses evident 12 weeks after anesthesia and surgery and that circulating EVs may serve as potential biomarkers of late postoperative alterations, with possible implications for neuroinflammatory signaling.

## Figures and Tables

**Figure 1:**
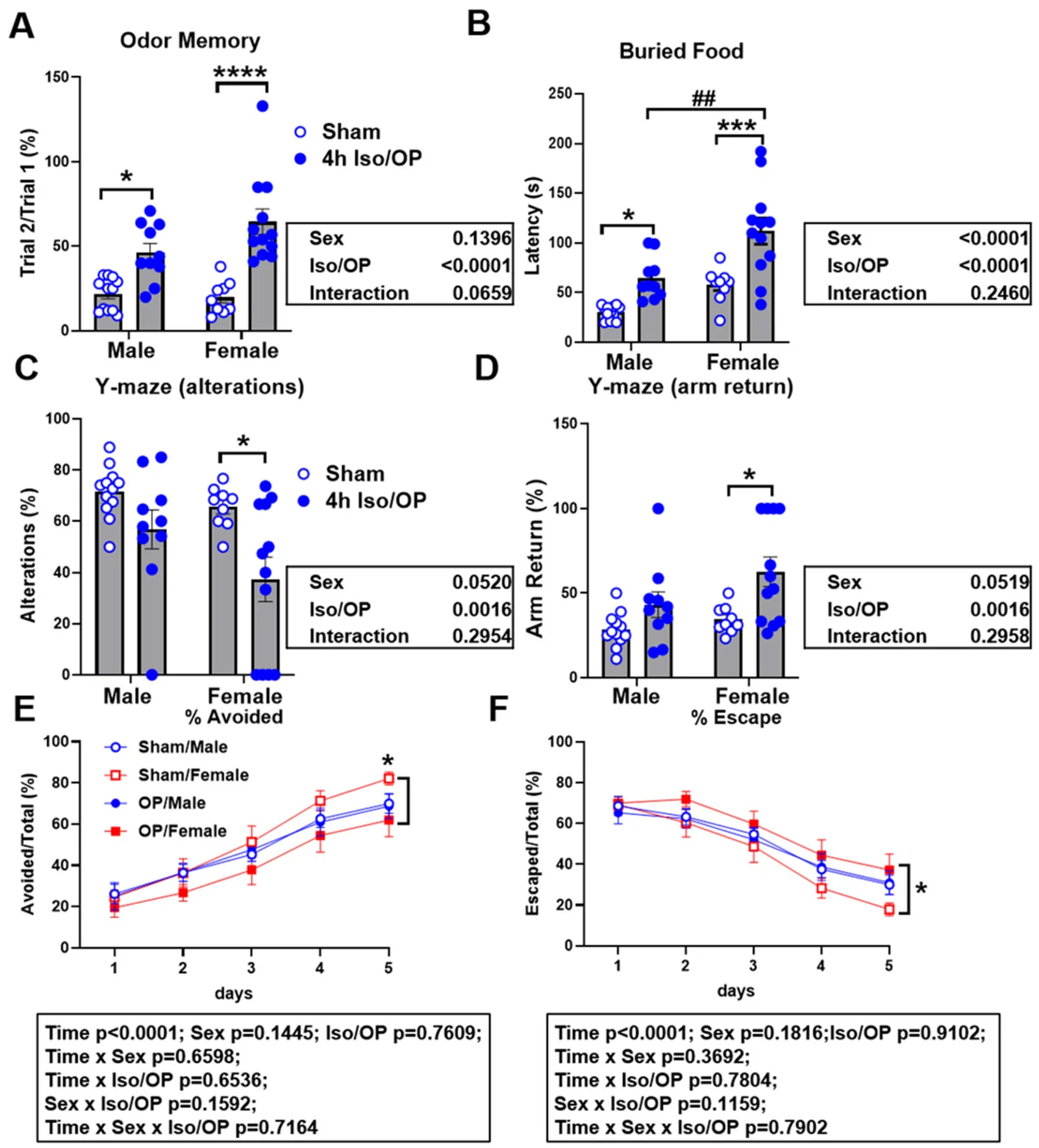
Four-hour isoflurane exposure with laparotomy induces chronic sex-dependent neurobehavioral deficits in adult C57BL/6 mice. Adult male and female mice (10–12 weeks old) were subjected to laparotomy under 4 h isoflurane exposure (Iso/OP), followed by behavioral analyses 3 months after exposure. (**A**,**B**) Olfactory function was assessed using the odor memory test (**A**) and buried food test (**B**). (**C**,**D**) Spatial working memory was assessed using the Y-maze test, including spontaneous alternations (**C**) and arm returns (**D**). (**E**,**F**) Active avoidance performance was assessed across 5 training days, including percentage avoided trials (**E**) and percentage escape trials (**F**), shown separately for male and female mice. *n* = 9–12 mice/group. * *p* < 0.05, *** *p* < 0.001, and **** *p* < 0.0001 vs. sham controls; ## *p* < 0.01 vs. Iso/OP male mice, as indicated. Data was analyzed by two-way ANOVA (**A**–**D**), and repeated-measures three-way ANOVA (**E**,**F**) with Tukey’s multiple-comparisons test. Two-way or three-way ANOVA *p* values for Sex, Iso/OP, and Sex x Iso/OP interaction are shown below each plot.

**Figure 2:**
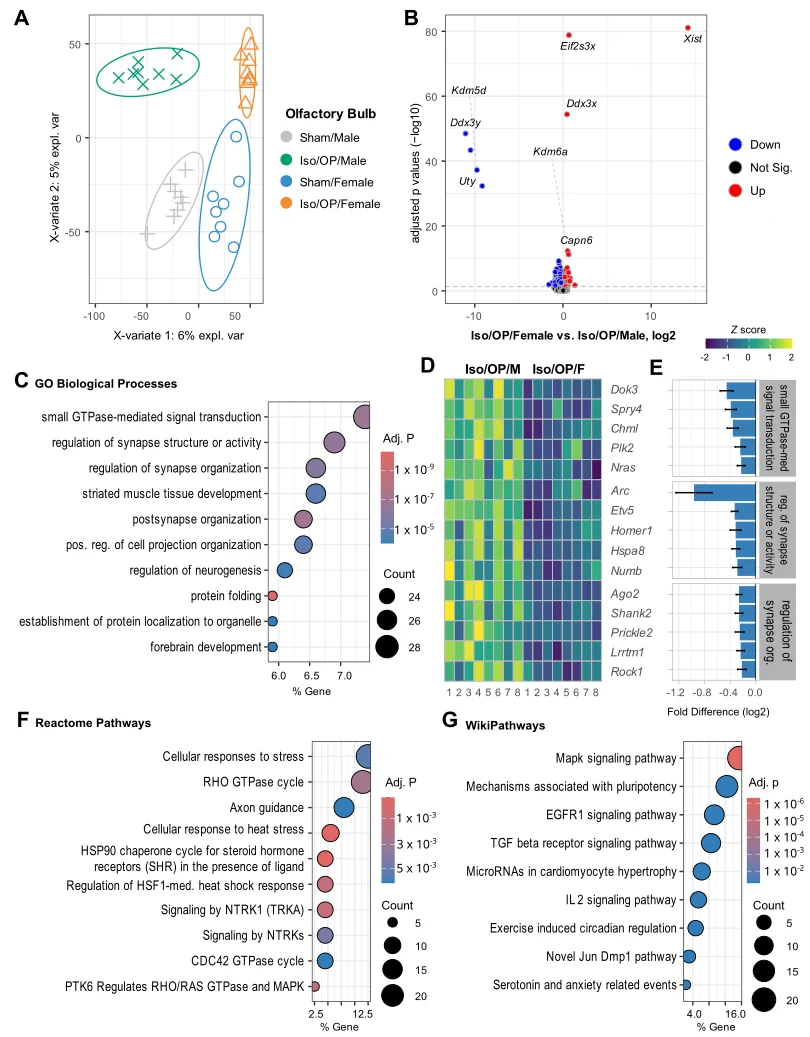
Four-hour isoflurane exposure with laparotomy induces sex-divergent transcriptomic remodeling in the olfactory bulb (OB) of adult mice. Adult male and female C57BL/6 mice (10–12 weeks old) were subjected to laparotomy under 4 h isoflurane exposure (Iso/OP), followed by euthanasia and OB dissection 3 months after exposure. (**A**) PLS-DA of normalized OB transcriptomic counts from sham and Iso/OP male and female mice. (**B**) Volcano plot of differentially expressed genes (DEGs) in Iso/OP/Female vs. Iso/OP/Male mice. (**C**–**E**) GO biological process enrichment of DEGs downregulated in Iso/OP/Female relative to Iso/OP/Male mice (**C**), with heatmap visualization of representative signature genes from selected enriched pathways (**D**) and fold-difference plots for representative pathway genes (**E**). (**F**,**G**) Reactome (**F**) and WikiPathways (**G**) enrichment analysis of downregulated DEGs in Iso/OP/Female relative to Iso/OP/Male mice. Dot size represents gene count, color indicates adjusted *p* value, and the x-axis shows gene ratio. RNAseq data were analyzed with DESeq2; *n* = 8 mice/group.

**Figure 3:**
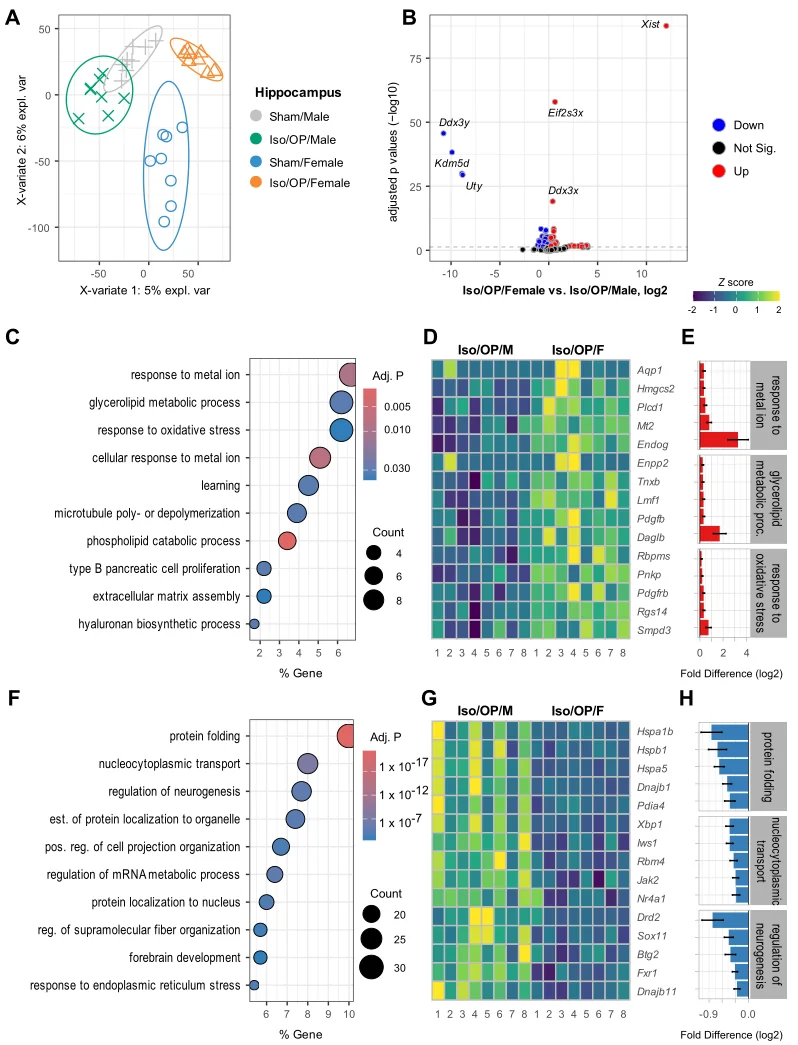
Four-hour isoflurane exposure with laparotomy induces sex-divergent transcriptomic remodeling in the hippocampus (HI) of adult mice. (**A**) PLS-DA of normalized HI transcriptomic counts from sham and Iso/OP male and female mice. (**B**) Volcano plot of DEGs in Iso/OP/Female vs. Iso/OP/Male mice. (**C**–**E**) GO biological process enrichment of DEGs upregulated in Iso/OP/Female relative to Iso/OP/Male mice (**C**), with heatmap visualization of representative signature genes from selected enriched pathways (**D**) and fold-difference plots for representative pathway genes (**E**). (**F**–**H**) GO biological process enrichment of DEGs downregulated in Iso/OP/Female relative to Iso/OP/Male mice (**F**), with heatmap visualization of representative signature genes from selected enriched pathways (**G**) and fold-difference plots for representative pathway genes (**H**). Dot size represents gene count, color indicates adjusted *p* value, and the x-axis shows gene ratio. RNAseq data were analyzed with DESeq2; *n* = 8 mice/group.

**Figure 4:**
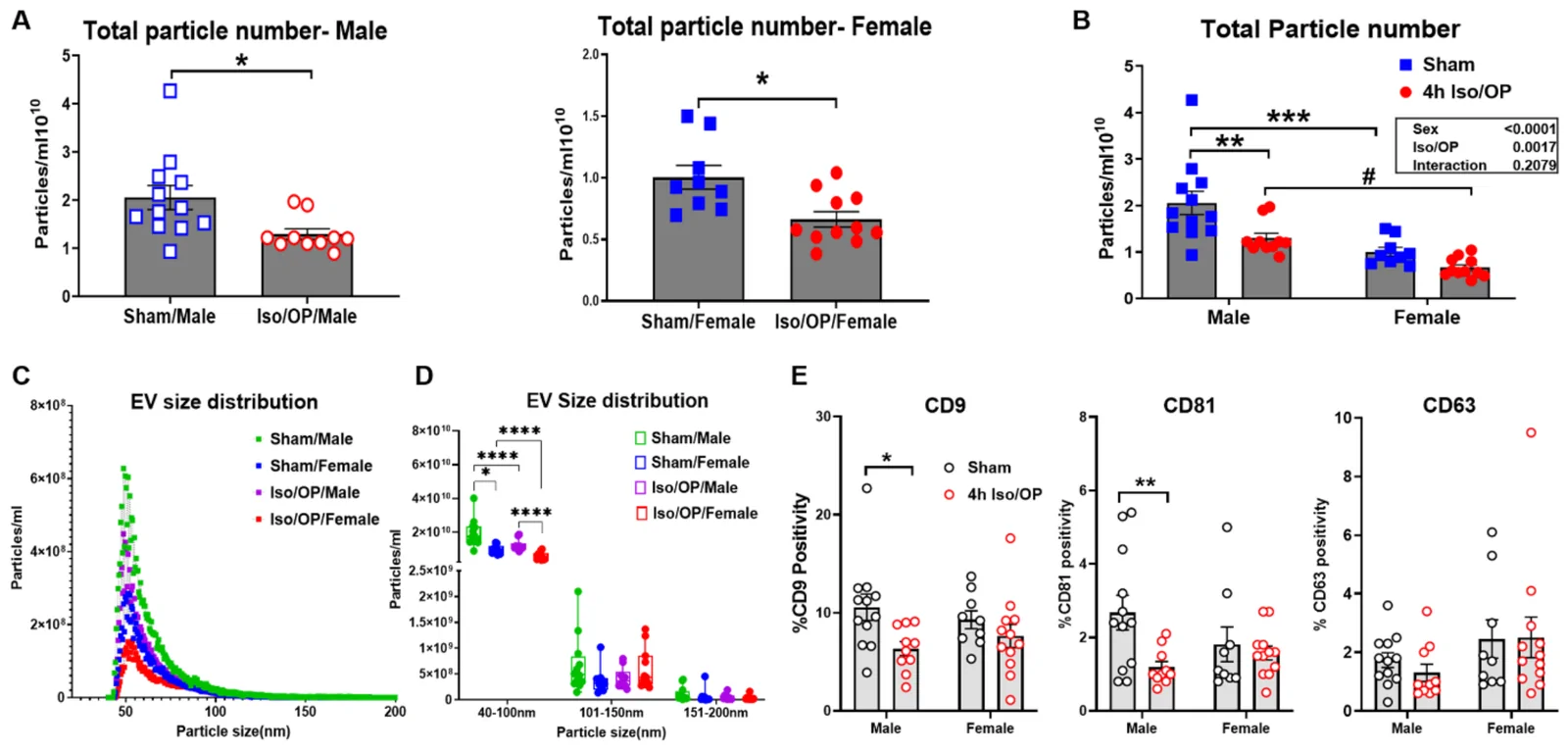
Four-hour isoflurane exposure with laparotomy alters circulating extracellular vesicle (EV) particle concentration and size distribution in young adult mice assessed 3 months after exposure. Plasma EV preparations were analyzed using NanoFCM. (**A**) Total particle concentrations in Sham and 4 h isoflurane/laparotomy (Iso/OP) groups, compared separately within males and females. (**B**) Total particle concentrations across sex and treatment groups. (**C**) Particle size distribution curves for each experimental group. (**D**) Particle concentrations within the indicated size ranges (40–100, 101–150, and 151–200 nm). (**E**) Percentages of particles positive for the EV-associated tetraspanins CD9, CD81, and CD63 in male and female Sham and Iso/OP groups. Bar graphs show mean ± SEM, with individual data points representing individual mice; *n* = 9–12 mice per group. Statistical analyses were performed using Student’s *t*-test (**A**) and two-way ANOVA followed by Tukey’s multiple-comparisons test (**B**,**D**,**E**). * *p* < 0.05, ** *p* < 0.01, *** *p* < 0.001, and **** *p* < 0.0001; # *p* < 0.05 for the indicated comparisons.

**Figure 5:**
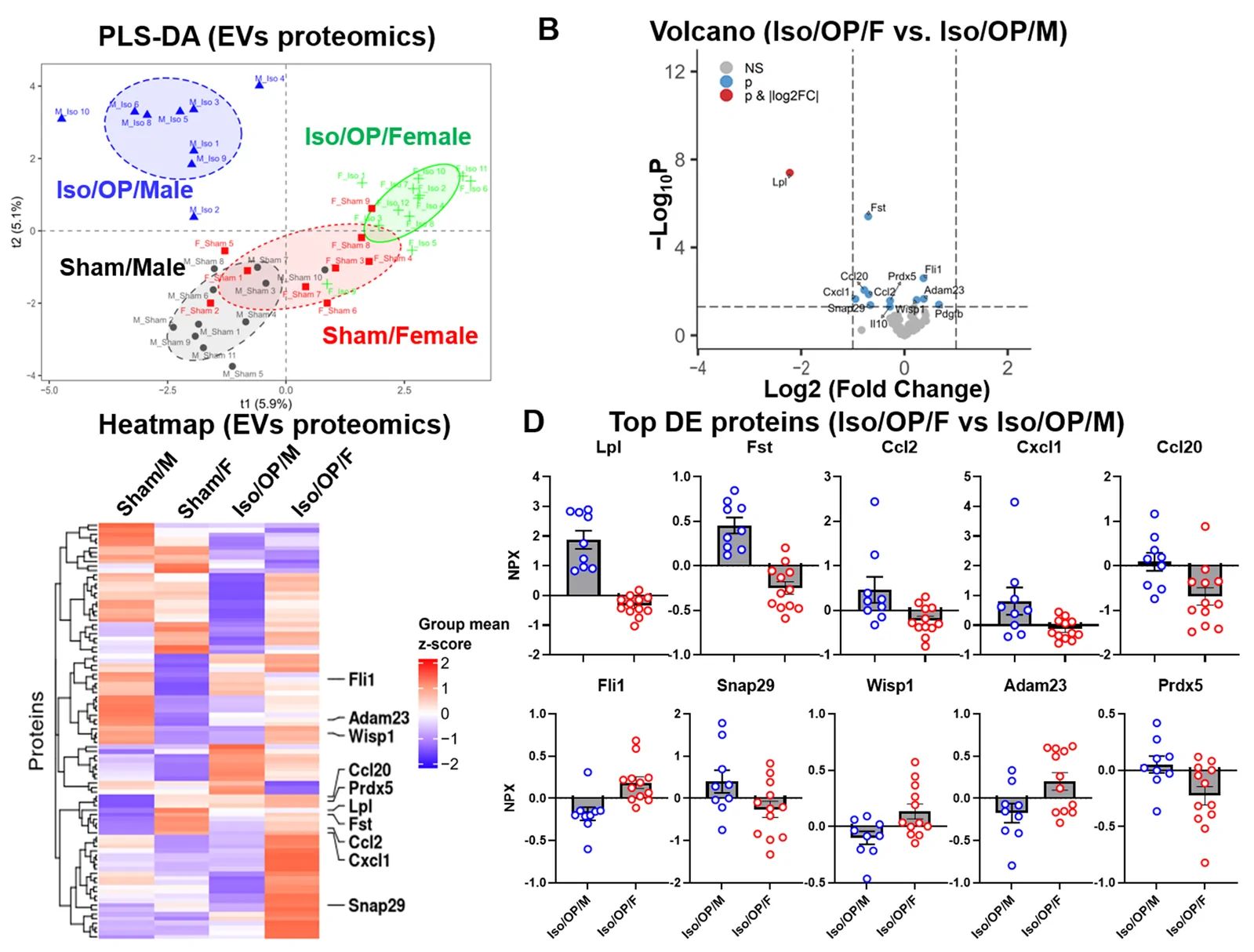
Circulating EV protein cargo is altered 3 months after 4 h isoflurane exposure with laparotomy in adult mice. Plasma EV protein cargo was profiled using the Olink Mouse Exploratory panel. (**A**) PLS-DA of EV proteomic profiles from sham and Iso/OP male and female mice. (**B**) Volcano plot of differentially represented EV proteins in Iso/OP/Female vs. Iso/OP/Male mice. Dashed lines indicate the nominal significance threshold (*p* = 0.05) and the prespecified effect-size threshold (|log2 fold change| = 1). (**C**) Group-mean z-score heatmap of EV protein abundance across experimental groups. (**D**) Individual abundance plots of representative differentially represented EV proteins between Iso/OP/Female and Iso/OP/Male mice. *n* = 9–12 mice/group.

**Figure 6:**
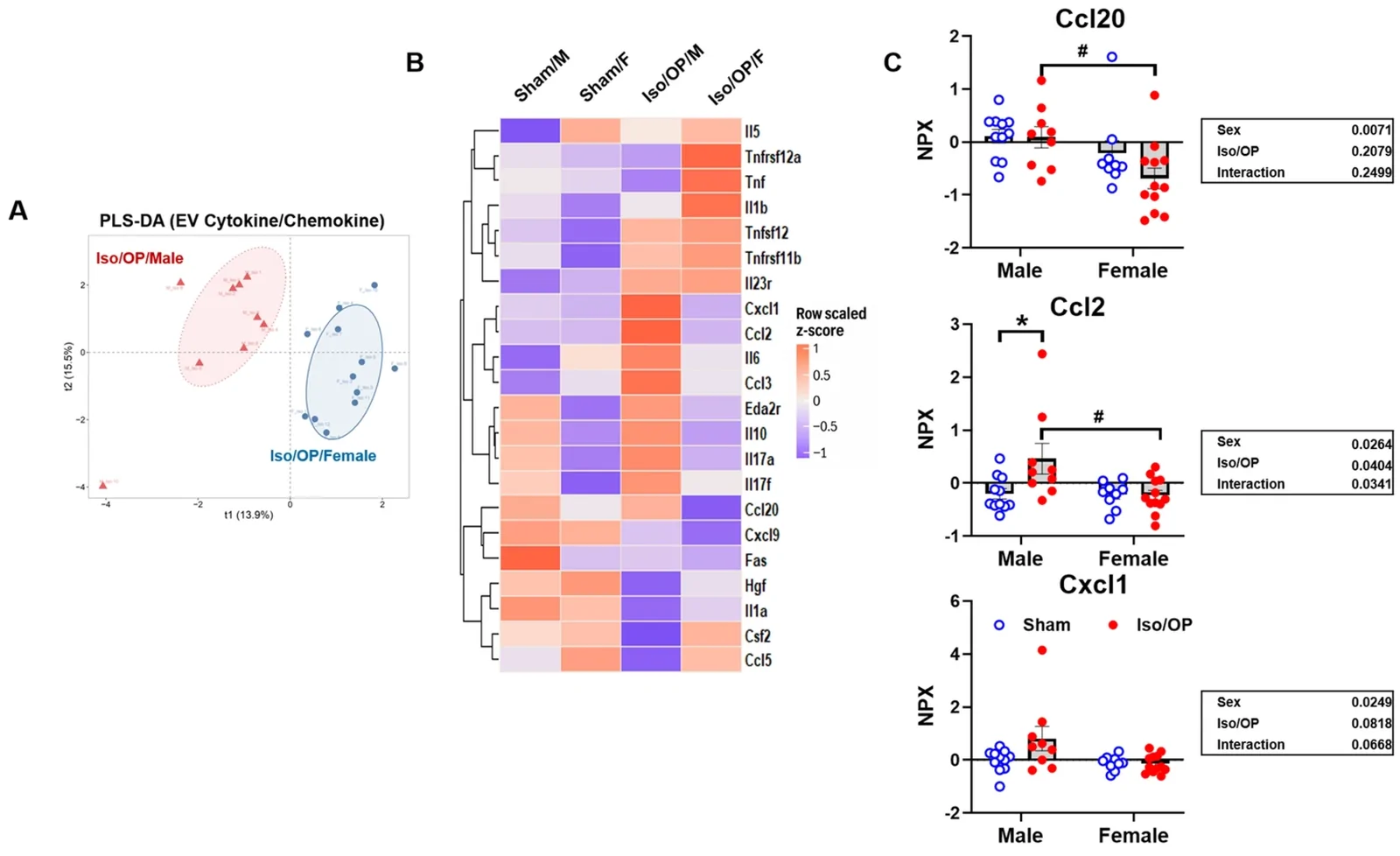
Iso/OP induces sex-divergent circulating EV cytokine and chemokine cargo profiles at 3 months after exposure. Circulating EVs were isolated from plasma collected 12 weeks after sham or Iso/OP exposure, and EV-associated inflammatory proteins were profiled using the Olink Mouse Exploratory panel. (**A**) PLS-DA score plot showing separation of Iso/OP male and Iso/OP female mice based on EV cytokine/chemokine cargo. Each point represents an individual animal; ellipses indicate group clustering. (**B**) Heatmap showing row-scaled z-scores of group-mean values for selected cytokine-, chemokine-, and inflammation-related EV cargo proteins across Sham/Male, Sham/Female, Iso/OP/Male, and Iso/OP/Female groups. Inflammation-related EV proteins were predefined based on KEGG annotations and published literature, independent of differential abundance or statistical significance. (**C**) Representative EV-associated inflammatory proteins showing sex- and Iso/OP-associated abundance patterns across experimental groups. Data are shown as individual animals with mean ± SEM. *n* = 9–12 mice/group.

**Figure 7:**
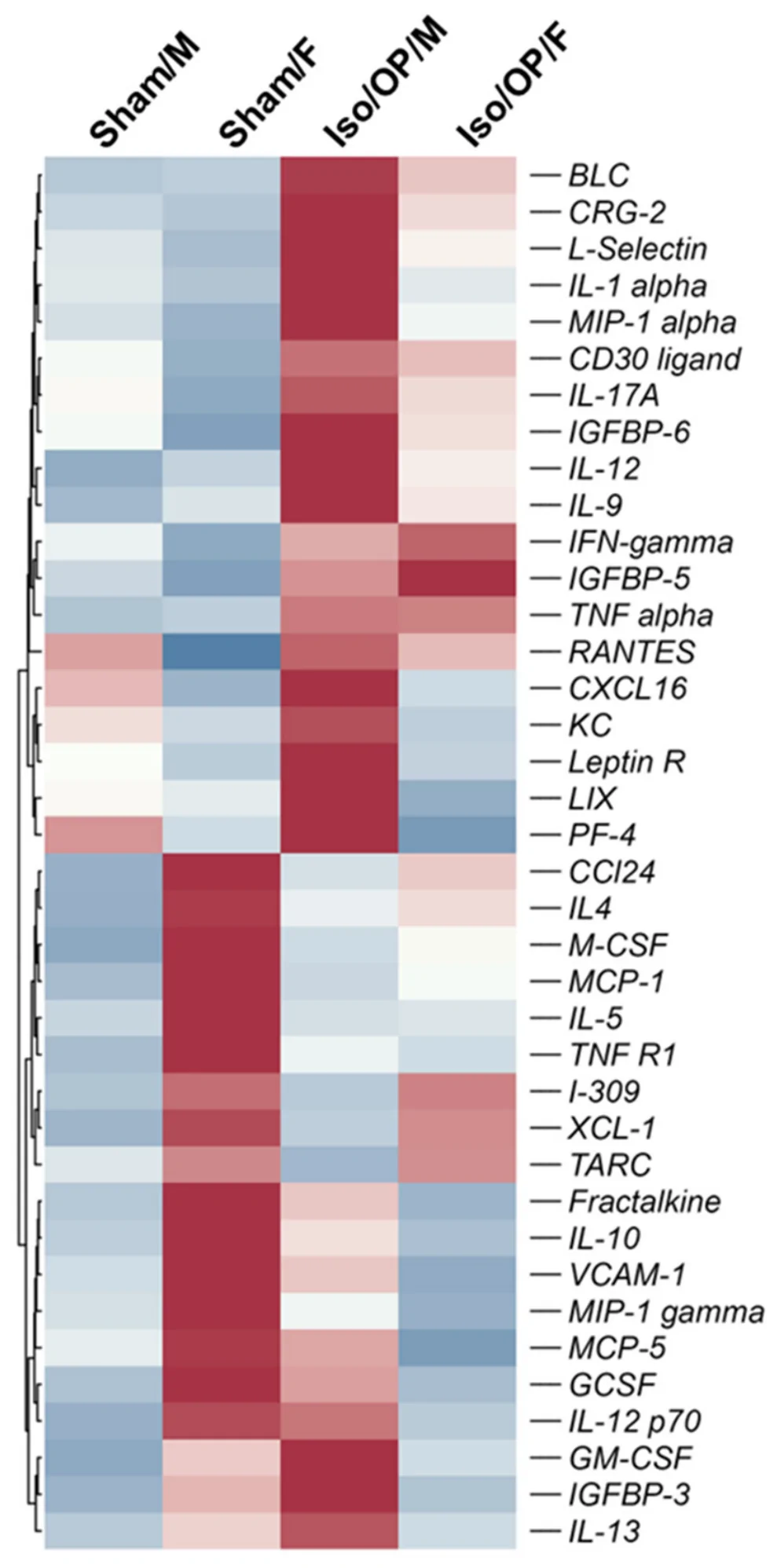
Hippocampal cytokine and chemokine profiles following injection of plasma EVs from male and female sham or Iso/OP donors. Plasma EVs were stereotactically injected into the hippocampus of naive adult male C57BL/6 recipient mice (5–6 recipients/group). Each recipient received 0.5 µL at each of two hippocampal sites at approximately 9 × 10^7^ measured particles/µL, corresponding to approximately 9 × 10^7^ particles per mouse. Hippocampal tissues were collected 24 h after injection and pooled within each recipient group, yielding one tissue pool per condition. Aliquots of the same pool were analyzed on two cytokine-array membranes, with duplicate spots per analyte on each membrane, yielding four technical measurements per analyte per condition. Spot intensities were normalized to positive control spots on each membrane and then to the male sham EV-injected group. Technical measurements were averaged within each condition before conversion to row-scaled z-scores for heatmap visualization. Analytes were clustered by relative abundance patterns. Results are descriptive and represent one pooled biological sample per condition.

**Figure 8:**
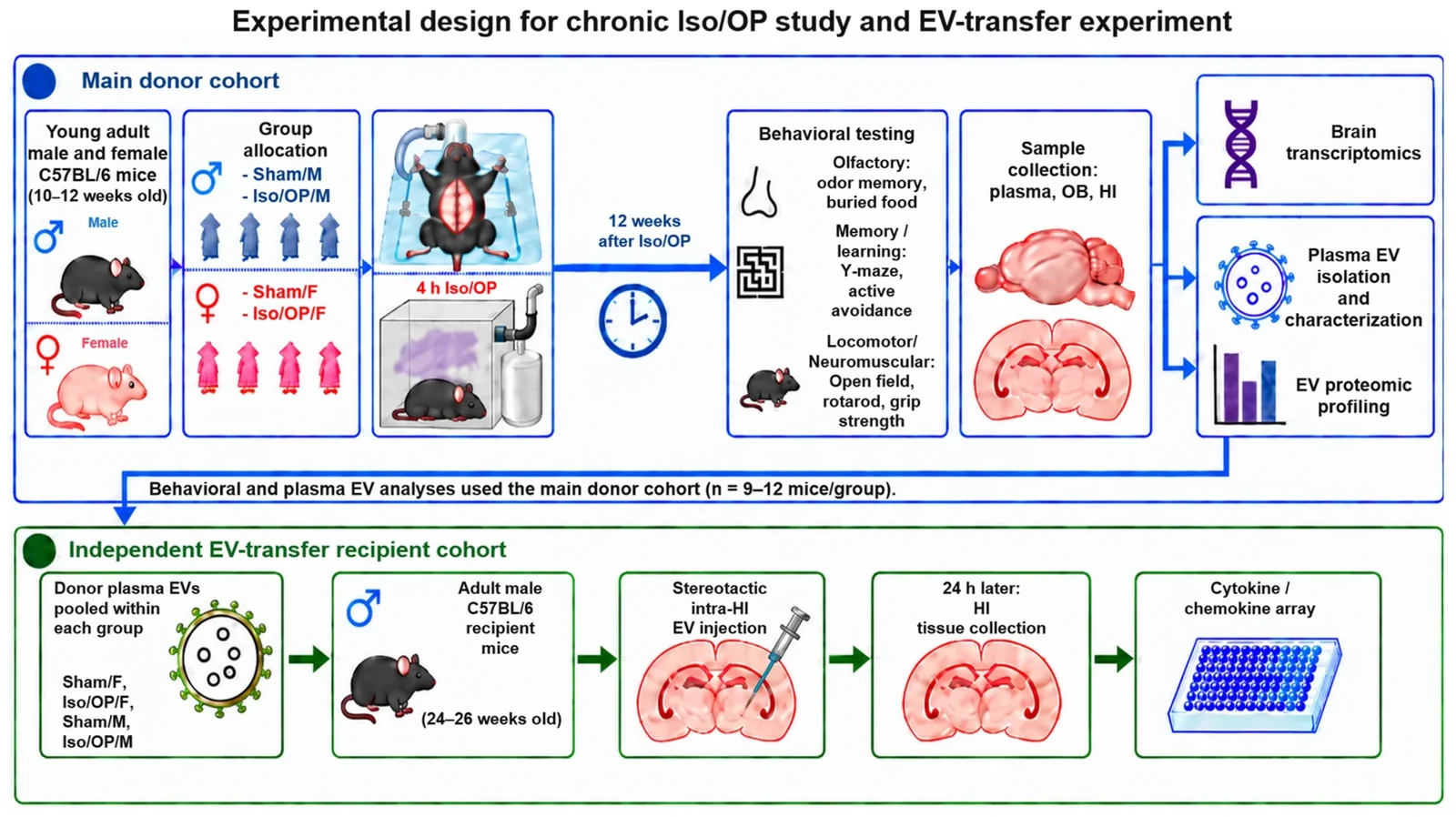
Experimental design for chronic Iso/OP study and EV-transfer experiment. Adult male and female C57BL/6 mice (10–12 weeks old) underwent sham treatment or 4 h of 2% isoflurane anesthesia combined with laparotomy (Iso/OP). Twelve weeks after exposure, mice were assessed using olfactory, cognitive, and neurological behavioral tests, including odor memory, buried food, Y-maze, active avoidance, open-field, rotarod, and grip strength assays. Plasma, olfactory bulb (OB), and hippocampal (HI) tissues were then collected for extracellular vesicle (EV) characterization, EV proteomic profiling, and brain transcriptomic analysis. Plasma EVs were quantified and sized by NanoFCM, EV-associated proteins were profiled using the Olink Mouse Exploratory panel, and OB and HI tissues were analyzed by RNAseq. In a separate EV-transfer experiment, plasma EVs pooled from each donor group were stereotactically injected into the hippocampus of naïve male recipient mice. Hippocampal cytokine and chemokine responses were measured 24 h later following plasma EV injection.

## Data Availability

The original contributions presented in this study are included in the article/Supplementary Material. Further inquiries can be directed to the corresponding author.

## Supplementary Materials

The following supporting information can be downloaded at: https://www.mdpi.com/article/10.3390/ijms27219936/s1.
