# Supplementary material for: Long-Term Effects of Isoflurane Anesthesia and Surgery: Sex-Associated Neurological Dysfunction and Alterations in Circulating Extracellular Vesicles
Source: Int J Mol Sci. 2026 Nov 6;27(21):9936. doi: 10.3390/ijms27219936 (PMC13764343; doi:10.3390/ijms27219936)
Supplement: Supplementary file 1 [file ijms-27-09936-s001.zip › Supplemental Files/Supple Material 1_additional figures 10-28-2026 yl.pdf]

Long-term effects of isoflurane anesthesia and surgery: Sex-associated neurological dysfunction and alterations in circulating extracellular vesicles

Yun Li[1a], Ruth Park[1ab], Balaji Krishnamachary[1], Hangnoh Lee[2], Zhuofan Lei[1], Hui Li[1], Junfang Wu[1,3]*

[1]Department of Anesthesiology and Shock, Trauma and Anesthesiology Research Center (STAR), University of Maryland School of Medicine, Baltimore, MD 21201, USA

[2]Department of Medicine, University of Maryland School of Medicine, Baltimore, MD 21201, USA

[3]University of Maryland-Medicine Institute for Neuroscience Discovery (UM-MIND), University of Maryland School of Medicine, Baltimore, MD 21201, USA

[a] Contributed equally to the manuscript.

[b] Ms. Ruth Park is a third-year medical student at University of Maryland School of Medicine, Baltimore, MD 21201, USA

*Corresponding author: Dr. Junfang Wu. Email: junfang.wu@som.umaryland.edu, phone: 410-706-5189.

Supplementary Information

Supplemental Information includes seven Supplemental figures and figure legends.

Figure S1

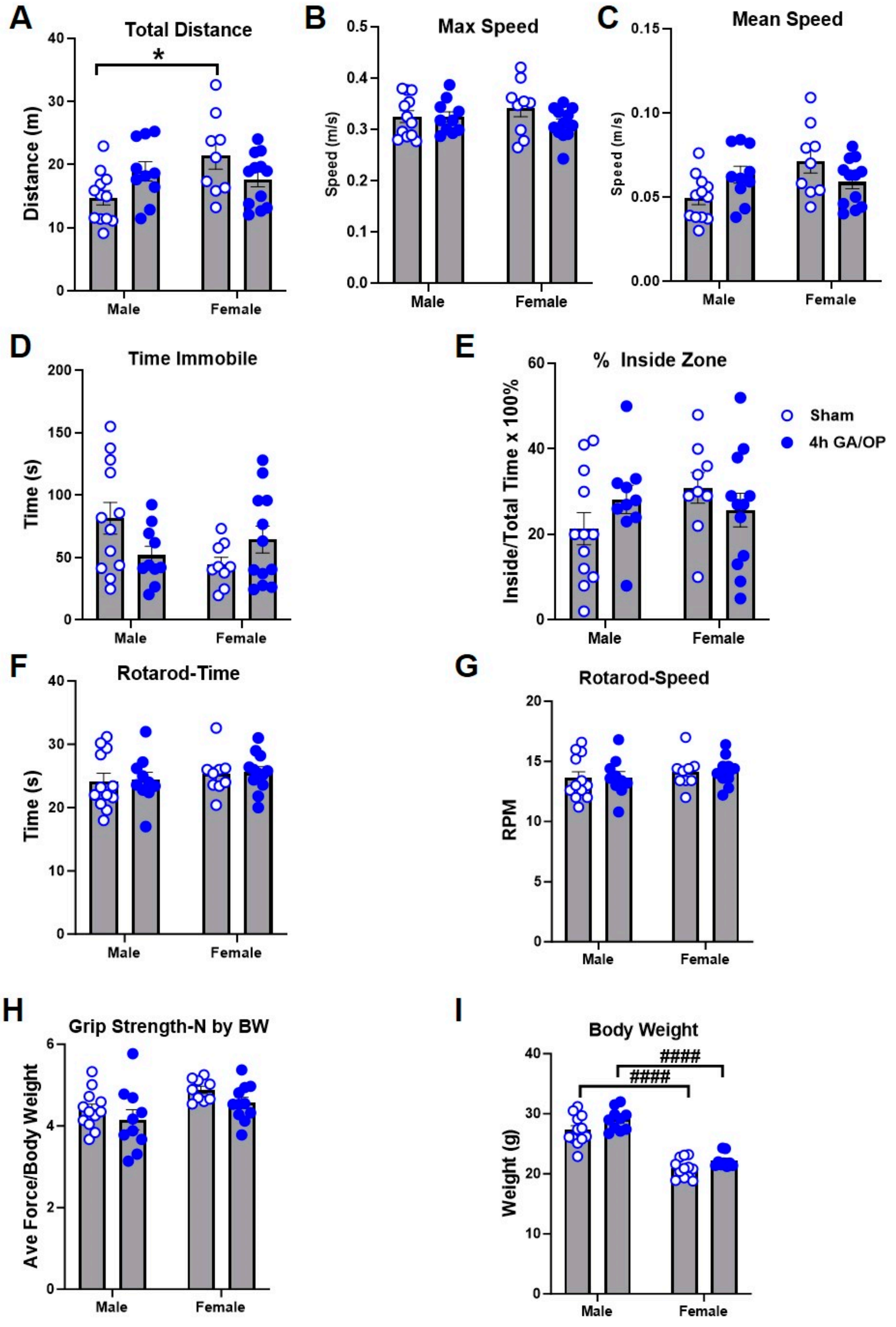

A
Total Distance
Distance (m)
B
Max Speed
Speed (m/s)
C
Mean Speed
Speed (m/s)
D
Time Immobile
Time (s)
E
% Inside Zone
Inside/Total Time x 100%
Sham
4h GA/OP
F
Rotarod-Time
Time (s)
G
Rotarod-Speed
RPM
H
Grip Strength-N by BW
Ave Force/Body Weight
I
Body Weight
Weight (g)
####
####
Male
Female

Figure S1. Locomotor activity, motor function, grip strength, and body weight following 4 h isoflurane and surgery. Male and female mice were assessed at 3 months following Sham or 4 h isoflurane and surgery (Iso/OP). **(A-E**) Open-field activity was evaluated by total distance travelled **(A)**, maximum speed **(B)**, mean speed **(C)**, time spent immobile **(D)**, and percentage of total time spent within the inside zone **(E)**. **F-G** Motor coordination was assessed by rotarod test, as recorded by latency to fall **(F)** from the accelerating rotarod and rotarod speed at fall **(G)**. **(H)** Grip strength was normalized to body weight. **(I)** Body weight of male and female mice. Open circles indicate Sham mice and filled circles indicate 4 h Iso/OP mice. Individual points represent individual animals, and bars represent mean ± SEM. *$p<0.05$; ####$p<0.0001$ for the indicated comparisons. Data were analyzed by two-way ANOVA with Tukey's multiple-comparisons test. n=9-12 mice/group.

Figure S2

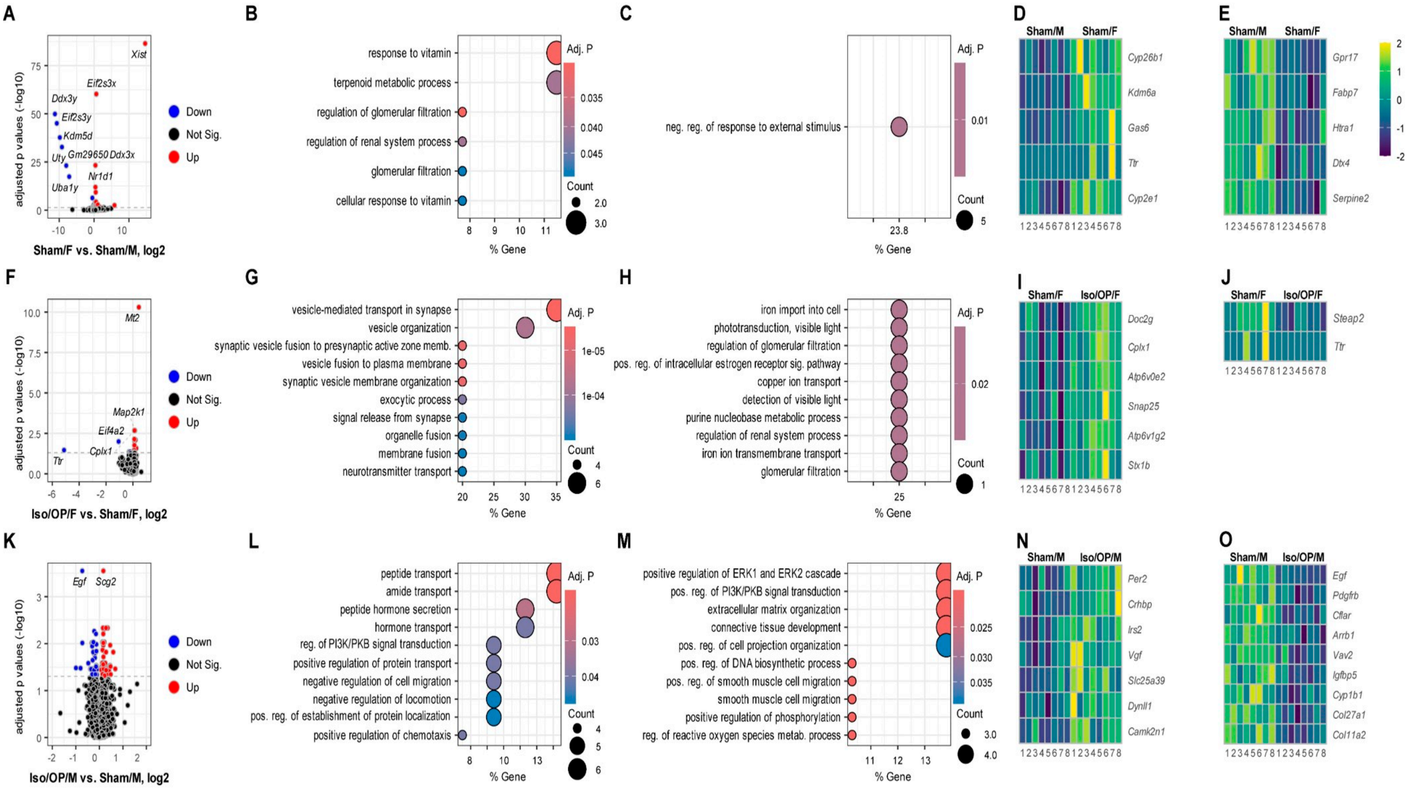

Figure S2. Baseline sex-associated differences and within-sex Iso/OP responses in OB transcriptomics at 3 months after exposure. **(A-E)** Baseline sex-associated differences in sham mice. Volcano plot showing DEGs in Sham/Female vs. Sham/Male mice **(A)**, GO biological process enrichment of upregulated and downregulated DEGs **(B-C)**, and heatmap visualization of representative genes from selected enriched pathways **(D-E)**. (**F-J**) Within-female Iso/OP response. Volcano plot showing DEGs in Iso/OP/Female vs. Sham/Female mice **(F)**, GO biological process enrichment of upregulated and downregulated DEGs **(G-H)**, and heatmap visualization of representative genes from selected enriched pathways **(I-J)**. (**K-O**) Within-male Iso/OP response. Volcano plot showing DEGs in Iso/OP/Male vs. Sham/Male mice **(K)**, GO biological process enrichment of upregulated and downregulated DEGs **(L-M)**, and heatmap visualization of representative genes from selected enriched pathways **(N-O)**. Dot size represents gene count, color indicates adjusted p value, and the x-axis shows gene ratio. RNA-seq data were analyzed with DESeq2; n=8 mice/group.

Figure S3

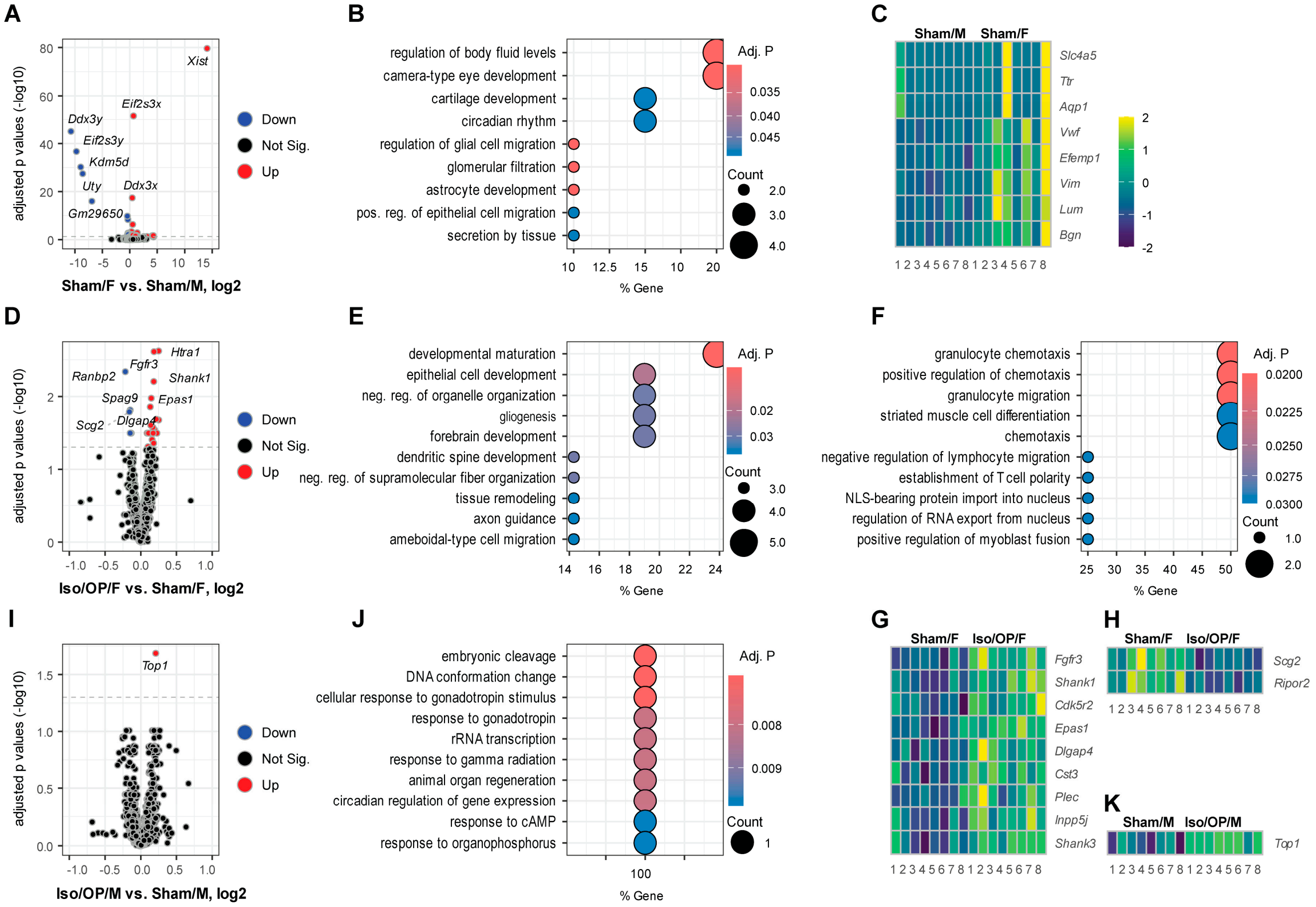

Figure S3. Baseline sex-associated differences and within-sex Iso/OP responses in HI transcriptomics at 3 months after exposure. (**A-C**) Baseline sex-associated differences in sham mice. Volcano plot showing DEGs in Sham/Female vs. Sham/Male mice **(A)**, GO biological process enrichment of baseline sex-associated DEGs **(B)**, and heatmap visualization of representative transcripts **(C)**. (**D-H**) Within-female Iso/OP response. Volcano plot showing DEGs in Iso/OP/Female vs. Sham/Female mice **(D)**, GO biological process enrichment of upregulated and downregulated DEGs **(E-F)**, and heatmap visualization of representative upregulated and downregulated genes **(G-H)**. (**I-K**) Within-male Iso/OP response. Volcano plot showing DEGs in Iso/OP/Male vs. Sham/Male mice **(I)**, GO biological process enrichment/annotation of male Iso/OP-associated DEGs **(J)**, and heatmap visualization of *Top1* expression across Sham/Male and Iso/OP/Male samples **(K)**. Dot size represents gene count, color indicates adjusted p value, and the x-axis shows gene ratio. RNA-seq data were analyzed with DESeq2; n=8 mice/group.

Figure S4

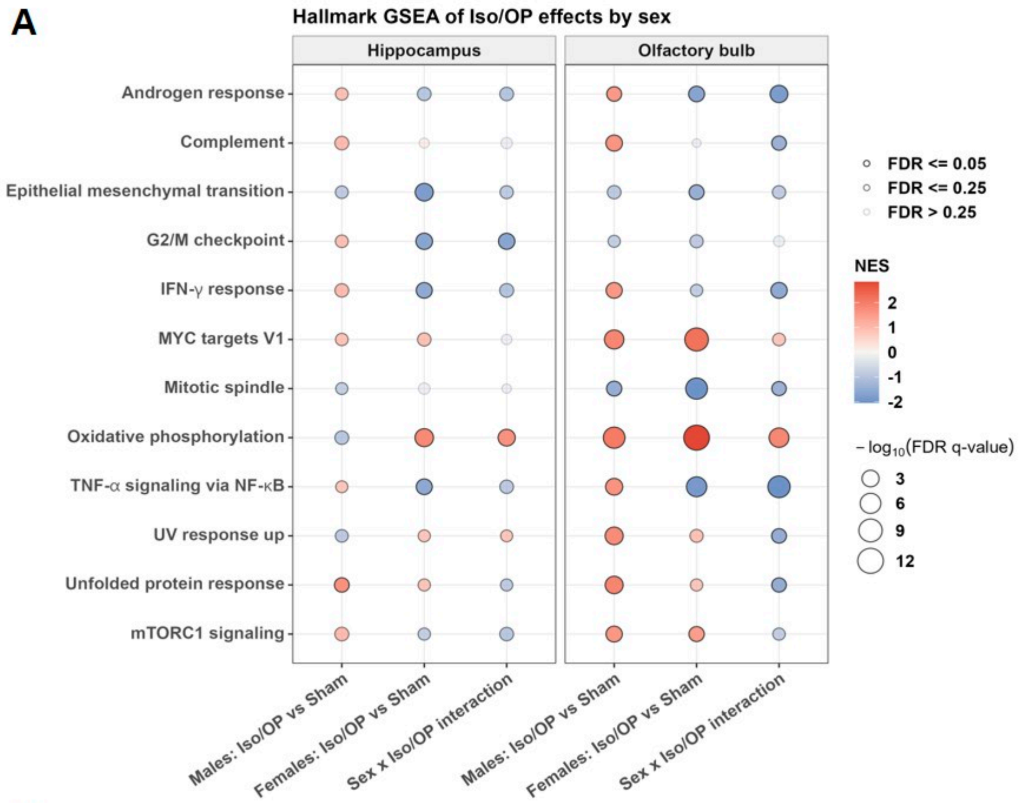
A
Hallmark GSEA of Iso/OP effects by sex
Hippocampus
Olfactory bulb
Androgen response
Complement
Epithelial mesenchymal transition
G2/M checkpoint
IFN-γ response
MYC targets V1
Mitotic spindle
Oxidative phosphorylation
TNF-α signaling via NF-κB
UV response up
Unfolded protein response
mTORC1 signaling
Males: Iso/OP vs Sham
Females: Iso/OP vs Sham
Sex x Iso/OP interaction
FDR <= 0.05
FDR <= 0.25
FDR > 0.25
NES
2
1
0
-1
-2
– log10(FDR q-value)
3
6
9
12

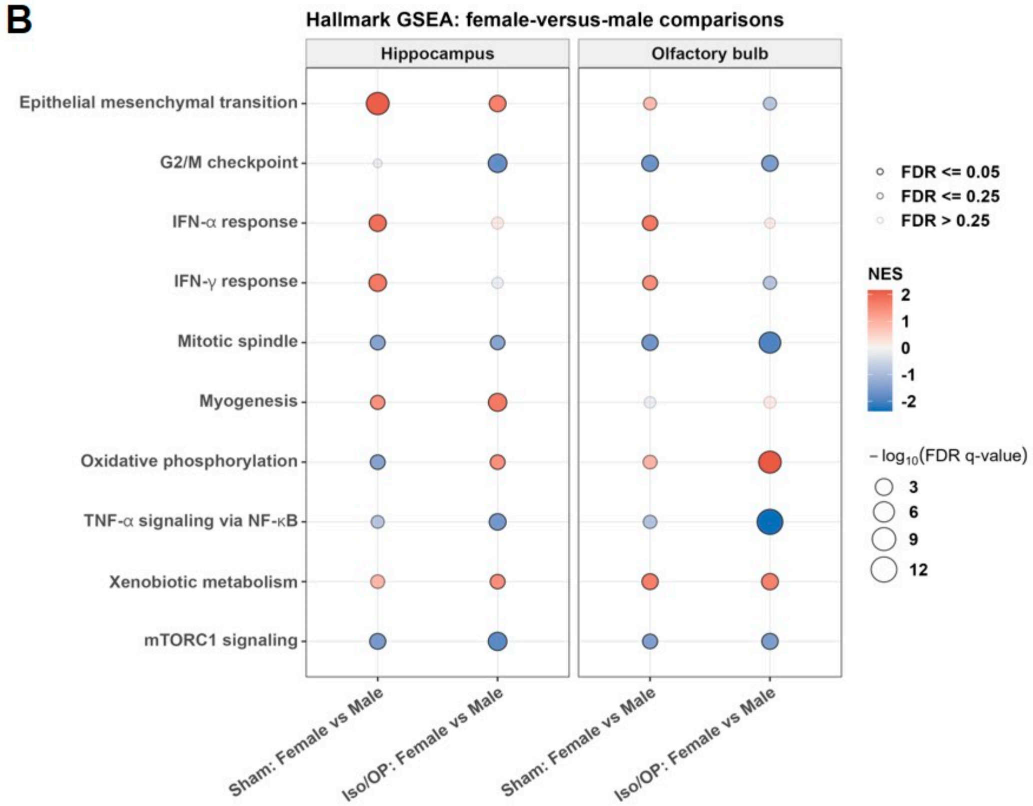
B
Hallmark GSEA: female-versus-male comparisons
Hippocampus
Olfactory bulb
Epithelial mesenchymal transition
G2/M checkpoint
IFN-α response
IFN-γ response
Mitotic spindle
Myogenesis
Oxidative phosphorylation
TNF-α signaling via NF-κB
Xenobiotic metabolism
mTORC1 signaling
Sham: Female vs Male
Iso/OP: Female vs Male
FDR <= 0.05
FDR <= 0.25
FDR > 0.25
NES
2
1
0
-1
-2
– log10(FDR q-value)
3
6
9
12

Figure S4. Gene set enrichment analysis of sex- and Iso/OP-dependent transcriptional responses in the hippocampus and olfactory bulb. Pre-ranked gene set enrichment analysis was performed using mouse Hallmark gene sets and the complete gene lists ranked by the DESeq2 Wald statistic. (**A**) Iso/OP-versus-sham enrichment in males and females and the Sex × Iso/OP interaction in the hippocampus and olfactory bulb. For the sex-stratified contrasts, positive normalized enrichment scores (NES) indicate enrichment after Iso/OP, whereas negative NES indicate enrichment in sham controls. For the interaction, positive NES indicate a stronger or more positive Iso/OP response in females relative to males, whereas negative NES indicate a stronger or more positive response in males relative to females. (**B**) Female-versus-male enrichment under sham and Iso/OP conditions. Positive NES indicate enrichment in females, and negative NES indicate enrichment in males. Pathways shown represent the union of the three highest-ranked pathways from each region-specific contrast, ordered by FDR q-value and absolute NES; corresponding enrichment results are displayed across all relevant comparisons. Circle color represents NES, circle size represents −log10(FDR q-value), and opacity indicates FDR significance thresholds. Complete GSEA results and leading-edge genes are provided in Table S1.

Figure S5

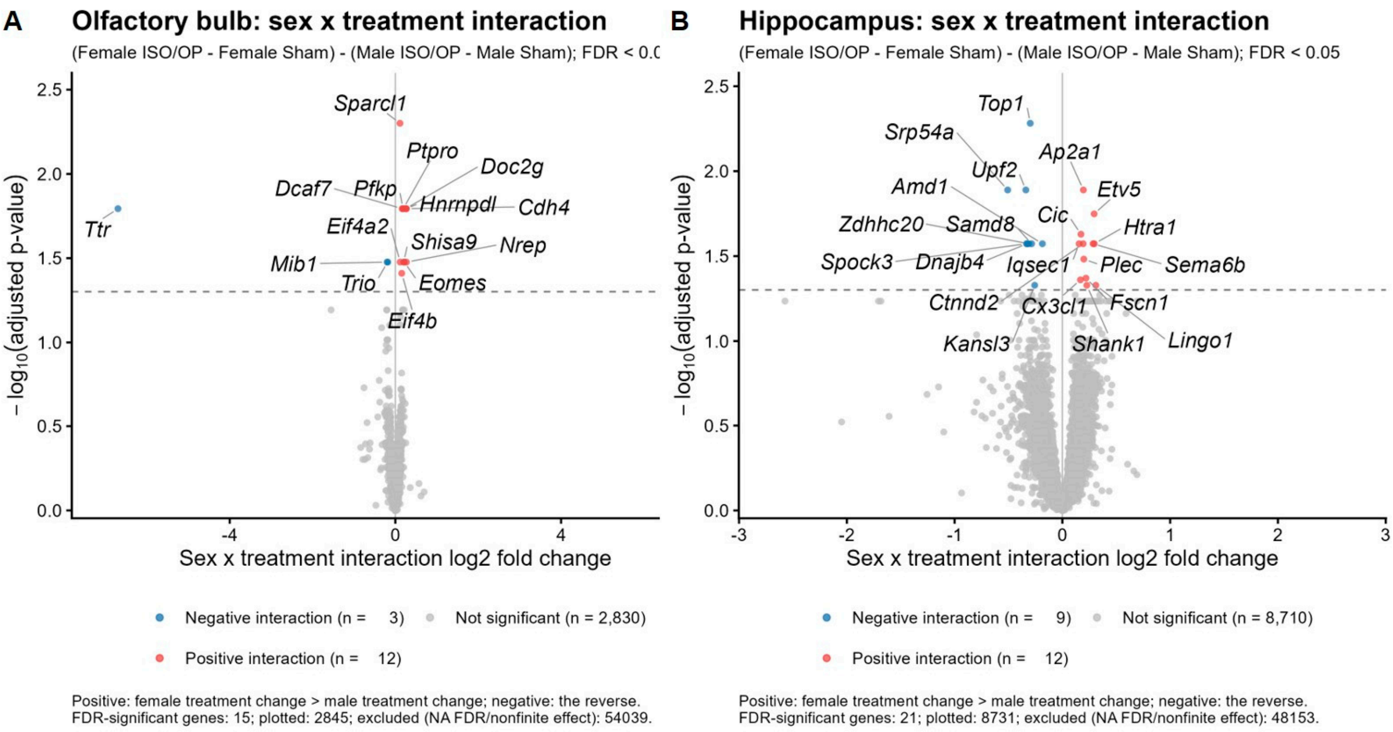

Figure S5. Sex × treatment interaction analysis of ISO/OP-associated transcriptomic changes in the olfactory bulb and hippocampus. Volcano plots showing sex × treatment interaction effects in the **(A)** olfactory bulb and **(B)** hippocampus regions. Interaction effects were calculated as the difference between the ISO/OP-associated log2 fold changes in female and male mice: (Iso/OP/Female – Sham/Female) – (ISO/OP/Male – Sham/Male). The x-axis represents the interaction log2 fold change, and the y-axis represents −log10 of the FDR-adjusted *p* value. Genes with significant positive interactions (red) exhibited greater treatment-associated expression changes in females relative to males, whereas genes with significant negative interactions (blue) exhibited the reverse. Gray points indicate genes without significant interaction effects. The horizontal dashed line indicates the FDR threshold of 0.05. Fifteen genes in the olfactory bulb and 21 genes in the hippocampus exhibited significant sex × treatment interactions (FDR < 0.05).

Figure S6

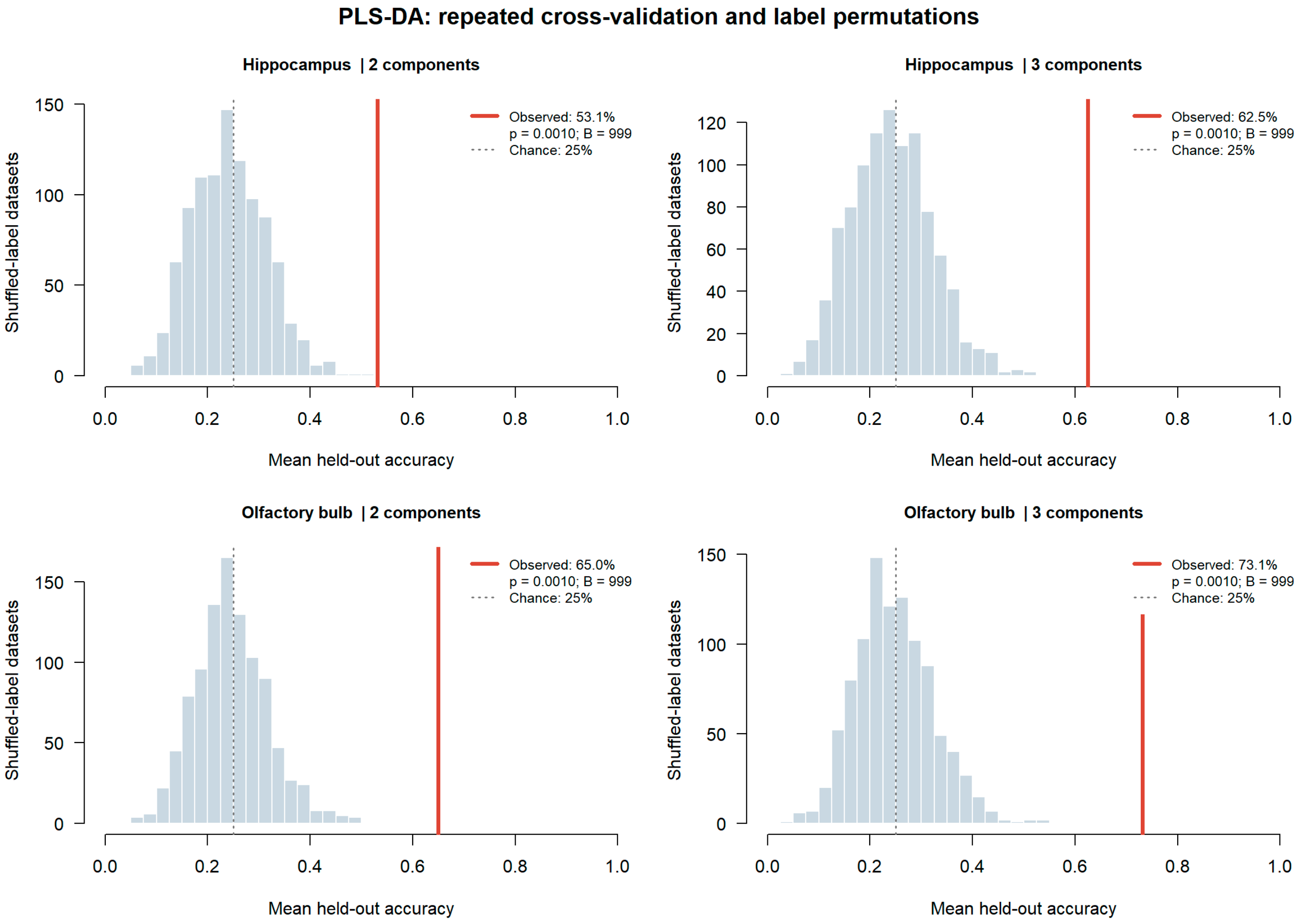

Figure S6. Internal validation of PLS-DA group separation. Histograms show the distribution of mean held-out classification accuracy across 999 label-permuted datasets for the hippocampus (top row) and olfactory bulb (bottom row), using two components (left) or three components (right). Each dataset was evaluated using five repeats of stratified four-fold cross-validation. Red lines indicate the observed mean accuracy, and dotted gray lines indicate nominal chance accuracy (25%). P-values are one-sided permutation p-values based on mean balanced error rate. All four experimental groups contained eight samples per region; repeated predictions do not represent additional biological replicates.

Figure S7

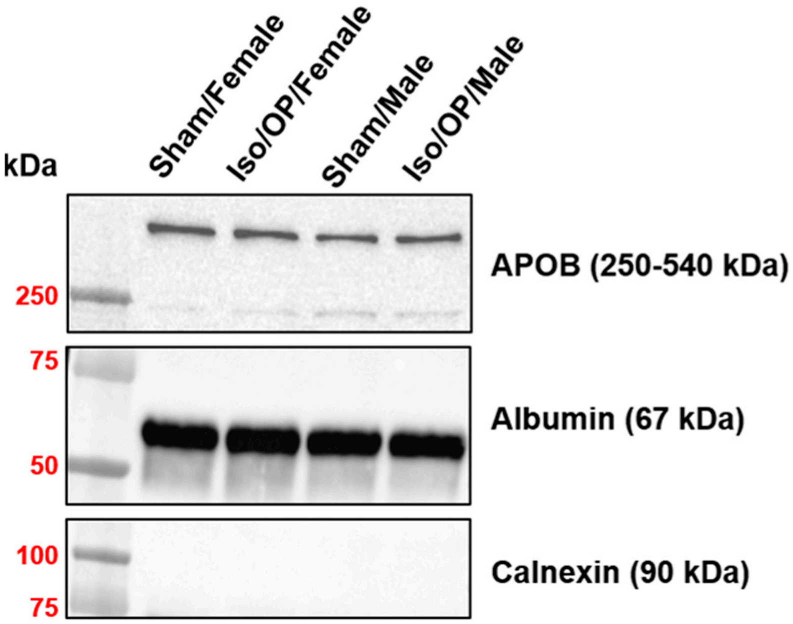

Figure S7. Immunoblot assessment of non-EV components in plasma extracellular vesicle (EV) preparations. Pooled plasma EV preparations from female and male Sham and 4 h isoflurane/laparotomy (Iso/OP) groups were analyzed by Western blotting for apolipoprotein B (ApoB), albumin, and calnexin (5 µg protein per lane). ApoB and albumin were detected, whereas calnexin was not detected under the assay conditions. Molecular weights are indicated in kDa.
